# Supplementary material for: The non-catalytic role of DNA polymerase epsilon in replication initiation in human cells
Source: Nat Commun. 2022 Nov 19;13:7099. doi: 10.1038/s41467-022-34911-4 (PMC9675812; doi:10.1038/s41467-022-34911-4)
Supplement: Supplementary file 1 — Supplementary Information [file 41467_2022_34911_MOESM1_ESM.pdf]

Supplementary Fig.1

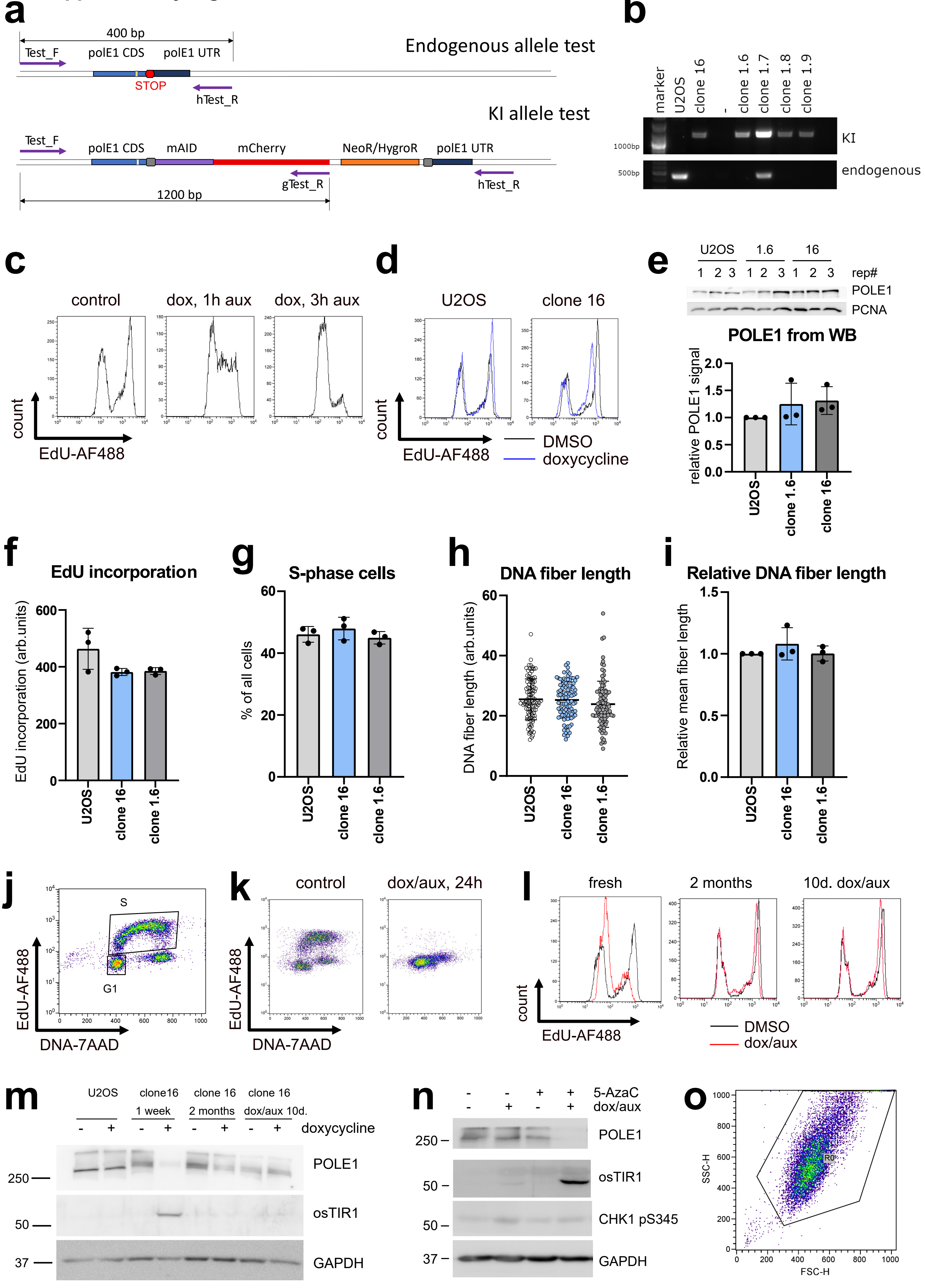

Supplementary Fig.1. Creating and characterizing a cell line expressing mAID-tagged polE1. a. Schematic representation of the PCR based testing of the knock-in efficiency. **b.** PCR testing the knock-in clones. Genomic DNA from control U2OS cells or indicated clonal lines was used as a template for PCR with primers Test_F and hTest_R (for endogenous allele) or Test_F and gTest_R (for knock-in allele). **c.** Clone 16 cells were treated for 16h with doxycycline, followed by 1h or 3h treatment with auxin, as indicated. EdU was added for the last 30 min. Flow cytometry analysis of EdU incorporation is shown. **d.** U2OS, clone 16 were treated for 24h with doxycycline. EdU was added for the last 30 min. Flow cytometry analysis of EdU incorporation is shown. **j.** Gating for G1 and S-phase populations. **k.** Homozygous mAID-KI clone 1.6 cells were treated for 24h with DMSO or dox/aux, 10µM EdU was added for the last 30 min of treatment**.** Flow cytometry plots showing EdU incorporation and DNA content (7-AAD staining) are shown. **l-m.** Clone 16 cells: fresh, cultured for 2 months without dox/aux, or cultured for 2 weeks with dox/aux were treated with DMSO or dox/aux for 16h. **l.** 10µM EdU was added for the last 30 min of treatment**.** Flow cytometry plots showing EdU incorporation are shown. **m.** Western blots of the total cell lysates are shown. **n.** Clone 16 cells cultured for 2 months were treated with 5-AzaC for 48h, dox/aux or DMSO were added for the last 16h of treatment where indicated. Western blots of the total cell lysates are shown. **o.** General gating strategy for the flow cytometry experiments. Source data are provided as a Source Data file.

Supplementary Fig. 2

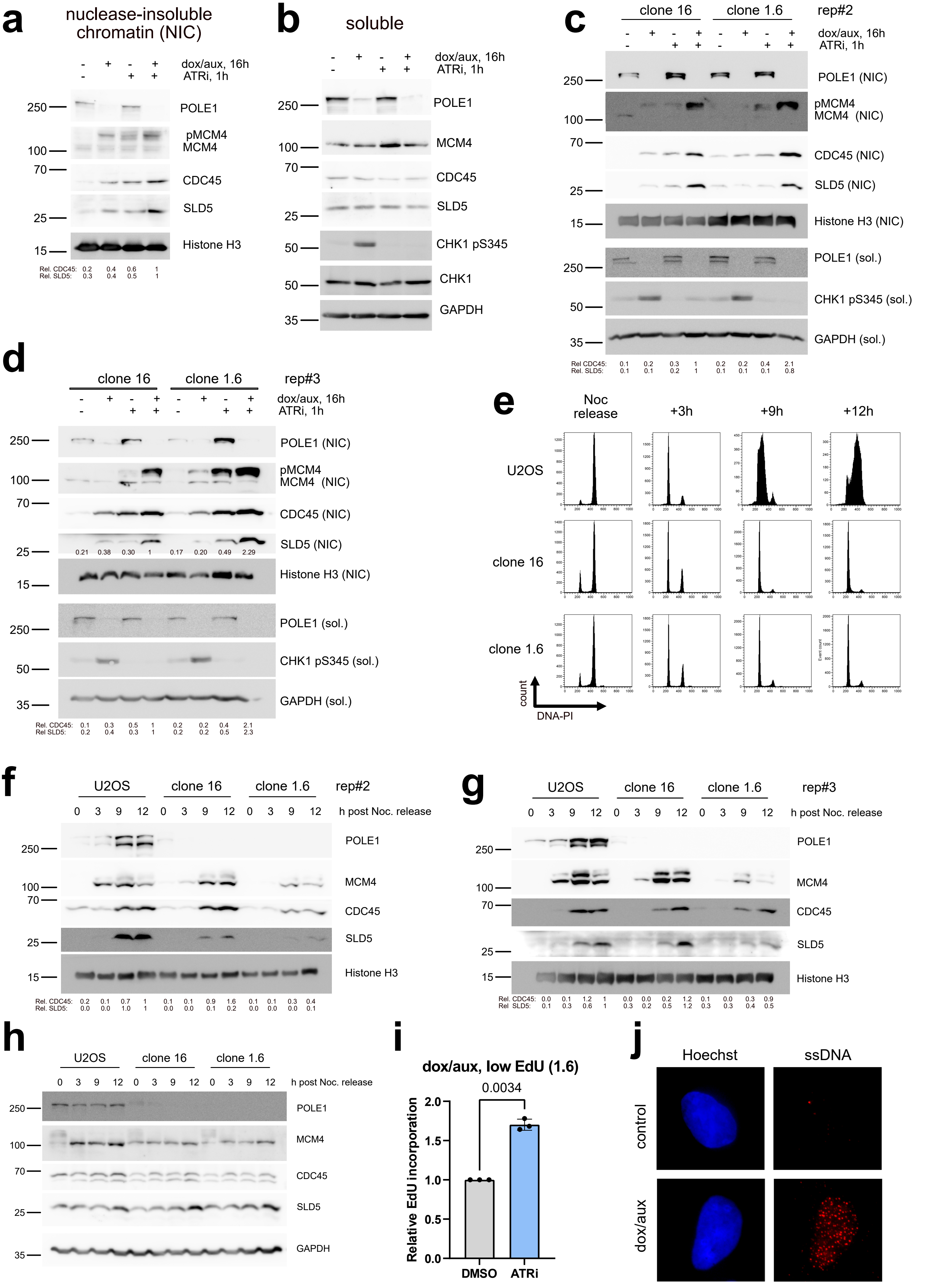

Supplementary Fig.2. Origin firing in POLE1-depleted cells. a-d. Homozygous mAID-KI clones 1.6 and/or clone 16 cells were treated for 16 h with DMSO or dox/aux, 5µM ATRi was added to the indicated samples for 1 h, followed by cell lysis and the isolation of the insoluble chromatin fraction. Western blot of nuclease-insoluble chromatin fraction (a, c, d – independent repeats) and soluble lysates (b) are shown. Equal amounts of protein were loaded. Specific signals of SLD5 and CDC45 were quantified by Fiji/ImageJ. **e-g**. Indicated cell lines were synchronized by thymidine/nocodazole blocks and treated with dox/aux as indicated on Fig.2c. Cell cycle analysis by PI staining (e) and western blot analysis of the chromatin fraction (f, g – second and third repeat) or soluble fraction (h) from the cells collected at the indicated timepoints are shown. Equal amounts of protein were loaded. Specific signals of SLD5 and CDC45 were quantified by Fiji/ImageJ. **i.** Clone 1.6 cells were treated for 16 h dox/aux, DMSO or 5µM ATRi was added to the indicated samples for 60 min before harvest, 10µM EdU was added for the last 30 min of treatment. Relative EdU incorporation, normalized to the samples without ATRi, is shown - mean + SD from n=3 independent experiments. Paired t-test was used for statistical analyses, p value is shown. **j.** Clone 16 cells were incubated with 10µM CldU for 48 h, DMSO or dox/aux were added for the last 16 h of treatment. After CSK extraction, cells were fixed and stained with anti-CldU antibodies under native conditions. Representative microscopy images are shown. Source data are provided as a Source Data file.

Supplementary Fig. 3

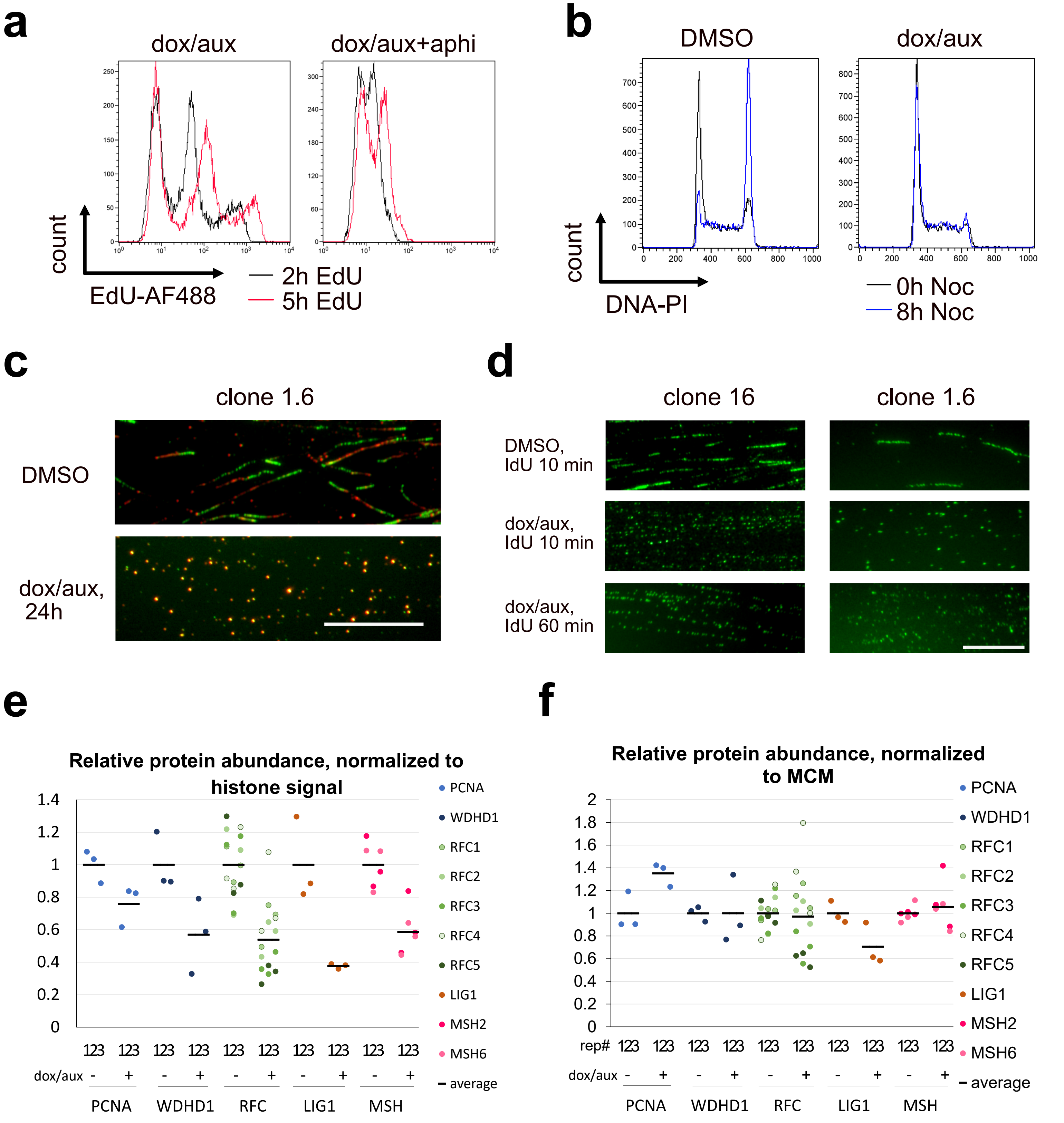

Supplementary Fig.3. The effect of POLE1 depletion on DNA synthesis. a. Clone 16 cells were treated for 16h with dox/aux, 10μM EdU was added for the indicated times before harvest, followed by ethanol fixation. 2μM aphidicolin was added 1h before the start of the EdU pulses where indicated. Flow cytometry plots showing EdU incorporation are shown. b. Clone 16 cells were treated for 16h with DMSO or dox/aux, followed by 8h nocodazole treatment where indicated. Flow cytometry plots of DNA content (PI) are shown. c. Clone 1.6 cells were treated for 24h with DMSO or dox/aux. Ongoing replication was labeled with 10 min pulse of CldU (red) followed by 10 min pulse of IdU (green) and visualized using DNA fiber analysis, as described in Methods. Scale bar is 20 μm. d. Clone 16 or 1.6 cells were treated for 24h with DMSO or dox/aux. Ongoing replication was labeled with a 10 or 60 min pulse of IdU and visualized using DNA fiber analysis, as described in Methods. Scale bar is 20 μm. e-f. Clone 16 cells were treated for 16h with DMSO or dox/aux, followed by 10 min EdU pulse and iPOND isolation of protein, associated with nascent DNA, and mass-spectrometry. The signal was normalized to average signal of histones (e) or MCM subunits (f) in each sample, and to respective DMSO-treated samples. The means from n=3 experimental replicates for each group are shown as horizontal black lines. Source data are provided as a Source Data file.

Supplementary Fig. 4

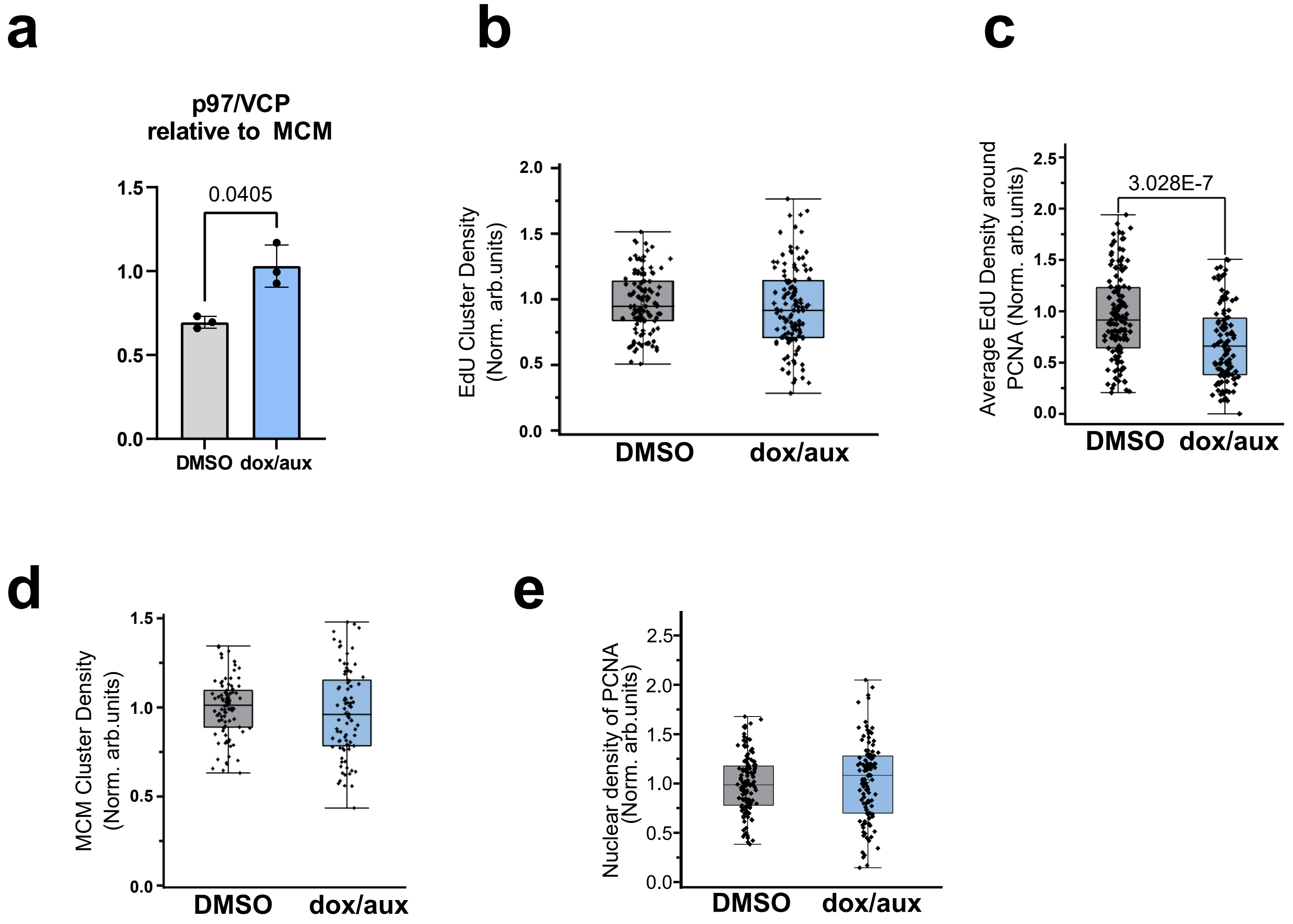

Supplementary Fig.4. MCM and PCNA on chromatin after POLE1 depletion. a. Clone 16 cells were treated for 16h with DMSO or dox/aux, followed by 10 min EdU pulse and iPOND isolation of protein, associated with nascent DNA, and mass-spectrometry. The signal was normalized to average signal of MCM subunits in each sample. Means + SD from n=3 independent experiments are shown. **b-e.** Clone 16 cells treated for 16 h with DMSO or dox/aux were pulse labeled with EdU for 15 minutes prior to processing for super-resolution imaging. Quantitation of number of EdU clusters (b), average density of EdU around PCNA (c) and number of MCM clusters (d) detected within a 6 × 6 μm^2 square region of interest, normalized to DMSO treated clone 16 cells based on at least 2 independent experiments. (For EdU cluster density, n= 118, 118; average EdU density around PCNA, n= 136, 109; MCM cluster density, n= 94, 86 for DMSO and dox/aux treated clone 16 cells). Quantitation of number of PCNA signal blinking detected within 6 × 6 μm^2 square region of interest, normalized to DMSO treated clone 16 cells based on at least 2 independent experiments. (n= 128, 121) (e). Mean + SD and the significant p values are shown (Student's t-test was used for statistical analyses). Source data are provided as a Source Data file.

Supplementary Fig. 5

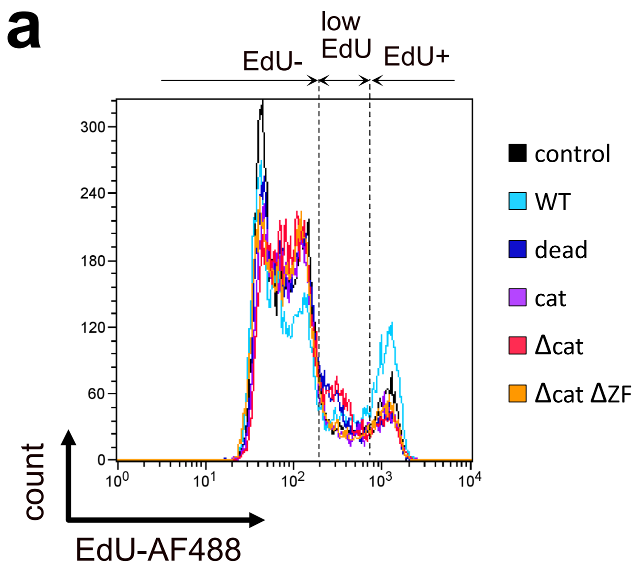

Supplementary Fig.5. Gating for the EdU flow cytometry experiments. a. Clone 16 cells were transfected with indicated constructs. 32h later dox/aux were added to the cells for 16h. 10μM EdU was added for the last 30 min. Flow cytometry histograms of the EdU channel and gating for “EdU-“, “low EdU” and “high EdU” fractions are shown.

Supplementary Fig. 6

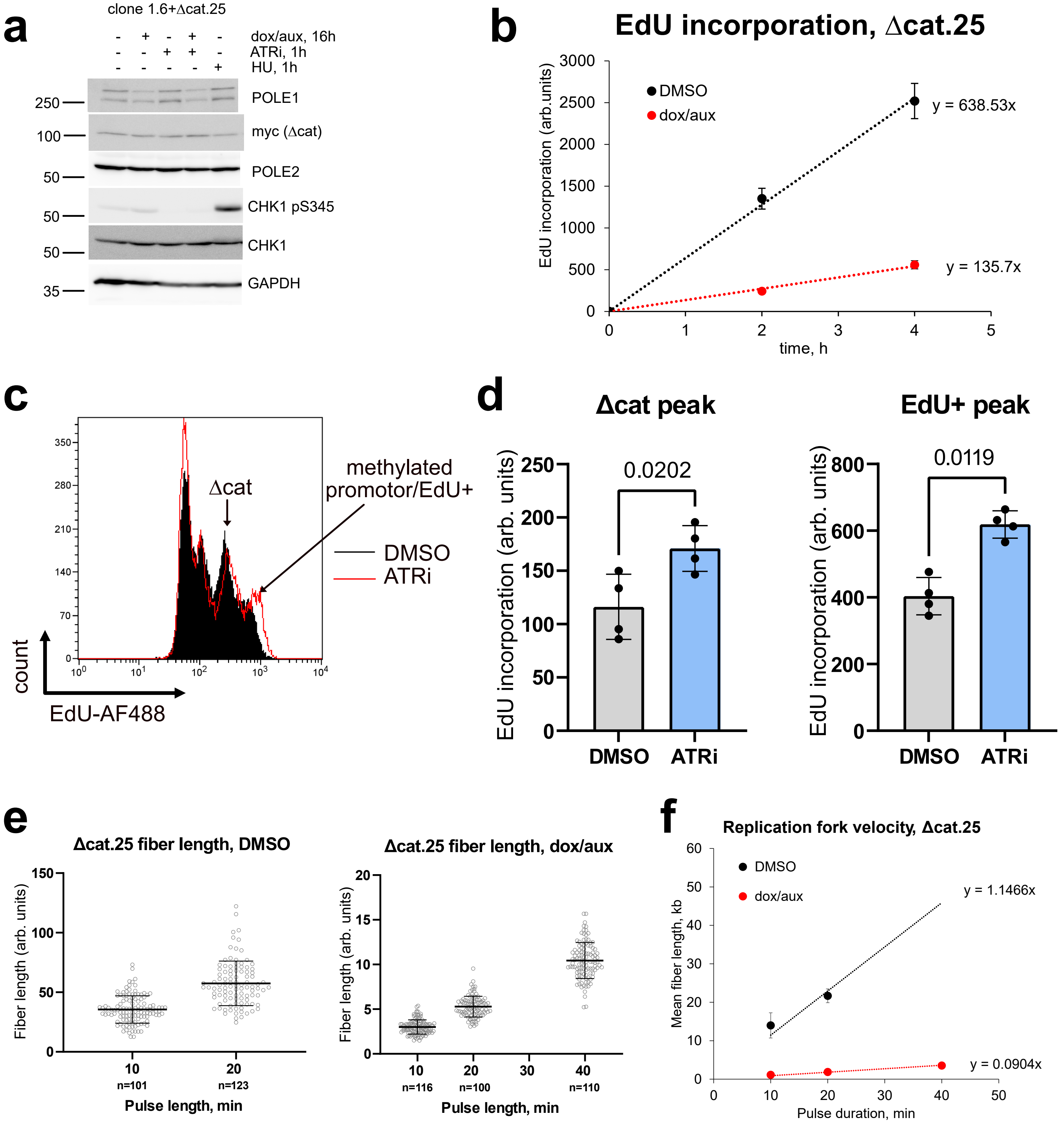

Supplementary Fig.6. DNA replication dependent on the C-terminal non-catalytic domain of POLE1. Clone 1.6 stably expressing myc-FLAG-Δcat - clone 25 (Δcat.25) were treated for 16 h with DMSO or dox/aux. a. 5μM ATRi was added to the indicated samples for 1 h. Western blot of the whole cell lysates is shown. b. EdU was added for last 2-4 h of treatment, EdU incorporation quantifications based on n=3 independent experiments, means + SD are shown, dox-resistant population was disregarded for quantification. c-d. Cells stably expressing myc-FLAG-Δcat (1.6+Δcat) were treated for 16h dox/aux, DMSO or 5 μM ATRi were added 15 min before the start for the 30 min EdU pulse. Flow cytometry histograms of EdU incorporation (c) or EdU incorporation quantifications (d) are shown. Quantification is based on n=4 independent experiments, means and standard deviations are shown, dox-resistant population was counted as EdU+, t-test was used for statistical analysis. e-f. Δcat.25 cells were treated for 16 h with DMSO or dox/aux. Ongoing replication was labeled with 10- or 40-min pulse of CldU followed by 20 min pulse of IdU and visualized using DNA fiber analysis, as described in Methods. Individual fiber lengths from a representative experiment (mean and SD) (e), and mean fiber lengths (based on n=3 experimental repeats) and SD of the means (f), are shown. Source data are provided as a Source Data file.

abundance	DMSO #1	DMSO#2	DMSO#3	dox/aux#1	dox/aux#2	dox/aux#3
**LIG1**	0.6256	0.544926	0.521031	0.51786015	0.34627732	0.3289423
**MCM2**	0.682898	0.847417	0.747545	0.7731149	0.62702406	0.6733367
**MCM3**	0.823308	0.788008	0.927816	0.3735569	0.48285681	0.767583
**MCM4**	0.980011	0.759096	0.947992	0.63777655	1.01983604	0.9909044
**MCM5**	0.942583	1.06148	0.951285	1.35494007	2.00810067	1.2255697
**MCM6**	1.060756	1.133231	0.718448	1.22006294	1.04787888	1.3228206
**MCM7**	1.510444	1.410768	1.706913	1.64054866	0.81430353	1.0197854
**MSH2**	5.560275	5.642784	5.51367	5.99351414	7.90505585	4.9217169
**MSH6**	6.457999	6.799287	7.846651	7.30308199	7.61321931	5.924885
**PCNA**	7.412415	9.78528	7.41678	11.6757068	11.4800534	10.118447
**POLA1**	0.6054	0.588787	0.509811	0.44749574	0.32924467	0.2709566
**POLA2**	0.182247	0.179608	0.214535	0.17475222	0.04647099	0.0162233
**POLD1**	1.589373	1.361132	1.767742	0.48225996	0.91745448	0.6068983
**POLD2**	0.370769	0.476797	0.487072	0.46147637	0.41631808	0.4503219
**POLD3**	0.307455	0.43055	0.423489	0.37018425	0.66230975	0.5478487
**POLE1**	0.268926	0.180554	0.246781	0	0.01162226	0
**POLE2**	0.061861	0.069378	0.061493	0	0	0
**RFC1**	1.821923	2.014611	1.990042	2.2393812	2.45445139	2.0310782
**RFC2**	1.574953	1.229896	1.718408	1.54697927	1.66850907	1.3671845
**RFC3**	2.041648	1.755711	2.605033	1.79453746	1.18756246	1.5071436
**RFC4**	1.925456	2.474083	3.158538	3.44298446	4.52240509	2.5222994
**RFC5**	1.724059	1.51033	1.420851	0.97089048	1.00646462	0.815534
**RPA1**	1.981708	2.05797	2.036621	2.62614043	1.92182318	1.5162494
**RPA2**	0.4537	0.326083	0.653788	0.38684571	0.08544443	0.2099663
**RPA3**	0.14799	0.193948	0.241255	0.16606404	0.07413179	0.0465284
**WDHD1**	1.054702	1.088423	0.957153	0.7942444	1.38407457	0.9229637
**HIST1H1**	21.50795	25.81274	25.57855	53.6741214	46.6646621	47.852836
**HIST1H2A**	27.81073	37.60954	33.60362	72.1429773	46.301193	51.740419
**HIST1H2B**	43.50907	58.56779	51.2026	134.822359	116.156248	91.93766
**HIST2H3A**	48.46719	73.01636	51.02598	163.925148	119.318694	80.829842
**HIST1H4A**	59.91248	82.30335	83.9806	131.265224	73.3093379	87.4093

Supplementary Table 1. Relative protein signal in the iPOND samples, normalized to the average signal of MCM in each sample. Does not represent relative protein abundance.
