# Supplementary material for: The non-catalytic role of DNA polymerase epsilon in replication initiation in human cells
Source: Nat Commun. 2022 Nov 19;13:7099. doi: 10.1038/s41467-022-34911-4 (PMC9675812; doi:10.1038/s41467-022-34911-4)
Supplement: Supplementary file 3 — Description of Additional Supplementary Files [file 41467_2022_34911_MOESM3_ESM.pdf]

Description of Additional Supplementary Files

File Name: Supplementary Data 1
Description: Summary of the iPOND data
