# Supplementary material for: The non-catalytic role of DNA polymerase epsilon in replication initiation in human cells
Source: Nat Commun. 2022 Nov 19;13:7099. doi: 10.1038/s41467-022-34911-4 (PMC9675812; doi:10.1038/s41467-022-34911-4)
Supplement: Supplementary file 5 — Reporting Summary [file 41467_2022_34911_MOESM5_ESM.pdf]

Corresponding author(s): Tatiana N. Moiseeva

Last updated by author(s): Nov 8, 2022

Reporting Summary

Nature Portfolio wishes to improve the reproducibility of the work that we publish. This form provides structure for consistency and transparency in reporting. For further information on Nature Portfolio policies, see our Editorial Policies and the Editorial Policy Checklist.

Statistics

For all statistical analyses, confirm that the following items are present in the figure legend, table legend, main text, or Methods section.

n/a	Confirmed	
☐	☒	The exact sample size (*n*) for each experimental group/condition, given as a discrete number and unit of measurement
☐	☒	A statement on whether measurements were taken from distinct samples or whether the same sample was measured repeatedly
☐	☒	The statistical test(s) used AND whether they are one- or two-sided<br>*Only common tests should be described solely by name; describe more complex techniques in the Methods section.*
☒	☐	A description of all covariates tested
☒	☐	A description of any assumptions or corrections, such as tests of normality and adjustment for multiple comparisons
☐	☒	A full description of the statistical parameters including central tendency (e.g. means) or other basic estimates (e.g. regression coefficient) AND variation (e.g. standard deviation) or associated estimates of uncertainty (e.g. confidence intervals)
☐	☒	For null hypothesis testing, the test statistic (e.g. *F*, *t*, *r*) with confidence intervals, effect sizes, degrees of freedom and *P* value noted<br>*Give P values as exact values whenever suitable.*
☒	☐	For Bayesian analysis, information on the choice of priors and Markov chain Monte Carlo settings
☒	☐	For hierarchical and complex designs, identification of the appropriate level for tests and full reporting of outcomes
☒	☐	Estimates of effect sizes (e.g. Cohen's *d*, Pearson's *r*), indicating how they were calculated

Our web collection on statistics for biologists contains articles on many of the points above.

Software and code

Policy information about availability of computer code

Data collection	for western blot imaging ImageQuant LAS4000 (V.1.2) software was used, for FACS data collection CellQuestPro (v0.3ef5b) was used, for fluorescent imaging with Olympus microscope CellSens Standard (v.1.14) was used, for SMLM Micro-Manager (v2.0) software was used. Quantification of western blots was performed using Fiji/ImageJ (version 1.53u).
Data analysis	For data analysis and plotting GraphPad Prism 9 and MS Excel were used, FACS data was initially processed using FACSalyzer.

For manuscripts utilizing custom algorithms or software that are central to the research but not yet described in published literature, software must be made available to editors and reviewers. We strongly encourage code deposition in a community repository (e.g. GitHub). See the Nature Portfolio guidelines for submitting code & software for further information.

Data

Policy information about availability of data

All manuscripts must include a data availability statement. This statement should provide the following information, where applicable:

- Accession codes, unique identifiers, or web links for publicly available datasets
- A description of any restrictions on data availability
- For clinical datasets or third party data, please ensure that the statement adheres to our policy

The mass spectrometry proteomics data generated in this study have been deposited to the ProteomeXchange Consortium (http://proteomecentral.proteomexchange.org) via the iProX partner repository 45 with the dataset identifier PXD033757. The UniProt Homo sapiens reference proteome data used in this study for mass-spectrometry data analysis are available in the Uniprot database under Proteome ID UP000005640. Source data are provided with this paper.

Field-specific reporting

Please select the one below that is the best fit for your research. If you are not sure, read the appropriate sections before making your selection.

☒ Life sciences ☐ Behavioural & social sciences ☐ Ecological, evolutionary & environmental sciences

For a reference copy of the document with all sections, see nature.com/documents/nr-reporting-summary-flat.pdf

Life sciences study design

All studies must disclose on these points even when the disclosure is negative.

Sample size	samples sizes were chosen as it is standard in the field and to allow sufficient sample sizes for statistical analyses:<br>15 000 cells/FACS experiment (for example, https://www.ncbi.nlm.nih.gov/pmc/articles/PMC3462567/)<br>>100 fibers/DNA fiber experiment (for example, https://star-protocols.cell.com/protocols/1626)<br>>50 cells for IF experiments (for example https://ejnmmires.springeropen.com/articles/10.1186/s13550-020-0604-8)
Data exclusions	No data were excluded from analyses unless clearly stated
Replication	All experiments were repeated at least 3-4 times as indicated in the figure legends, all replication attempts were successful
Randomization	n/a - no human or animal experiments were included in this study, randomization could not be applied to our cell line-based experiments
Blinding	the researchers were not blinded, as our methods of analyses have very low probability for a bias, and the differences between samples are too obvious

Reporting for specific materials, systems and methods

We require information from authors about some types of materials, experimental systems and methods used in many studies. Here, indicate whether each material, system or method listed is relevant to your study. If you are not sure if a list item applies to your research, read the appropriate section before selecting a response.

Materials & experimental systems

n/a	Involved in the study	
☐	☒	Antibodies
☐	☒	Eukaryotic cell lines
☒	☐	Palaeontology and archaeology
☒	☐	Animals and other organisms
☒	☐	Human research participants
☒	☐	Clinical data
☒	☐	Dual use research of concern

Methods

n/a	Involved in the study	
☒	☐	ChIP-seq
☐	☒	Flow cytometry
☒	☐	MRI-based neuroimaging

Antibodies

Antibodies used	POLE1 (Santa Cruz, #sc-390785, 1:500), osTIR1 (#PD048, 1:1000), GAPDH (Santa Cruz, #sc-47724, 1:1000), pCHK1 (Cell Signaling, #2360S, 1:500), Chk1 (Cell Signaling, #2348S, 1:1000), MCM4 (Cell Signaling, #3228S, 1:300), CDC45 (Santa Cruz, #sc-55569, 1:500), SLD5 (Santa Cruz, #sc-398784, 1:300), H3 (Santa Cruz, #sc-517576, 1:1000), MCM7 (Santa Cruz, #sc-9966, 1:1000), POLE2 (Santa Cruz, #sc-398582, 1:500), PCNA (Santa Cruz, #sc-56, 1:1000), FLAG (Sigma, F3165-1MG, 1: 3000) , myc (Cell Signaling, #2276, 1:1000).<br>Antibodies used for DNA fiber analysis:<br>BrdU Clone B44 (BD, #347580, 1:50), CldU (ab6326, 1:50), goat anti-mouse AlexaFluor 488 (Invitrogen, #A-11001, 1:150), goat anti-rat AlexaFluor 594 (Invitrogen #A-11007, 1:150)<br>Antibodies used for SMLM:<br>MCM6 (Abcam ab236151, 1:2500), PCNA (Santa Cruz, #sc-56, 1:1000), Goat anti-Mouse Alexa Fluor 488 (Invitrogen # A11029, 1:5000)
Validation	All antibodies are commercially available and were not additionally validated in this study.<br><br>POLE1 (Santa Cruz, #sc-390785) antibody was validated for WB on human cancer cells by the manufacturer:<br>https://www.scbt.com/p/dna-pol-epsilon-a-antibody-d-10<br>osTIR1 (#PD048) antibody is validated for WB on human cancer cells by the manufacturer:<br>https://www.mblbio.com/bio/g/dtl/A/?pcd=PD048<br>GAPDH (Santa Cruz, #sc-47724) antibody was validated for WB on human cancer cells by the manufacturer:<br>https://www.scbt.com/p/gapdh-antibody-0411<br>CHK1 (Cell Signaling, #2360S) antibody was validated for WB on human cancer cells by the manufacturer:<br>https://www.cellsignal.com/products/primary-antibodies/chk1-2g1d5-mouse-mab/2360

pCHK1 (Cell Signaling, #2348S) antibody was validated for WB on human cancer cells by the manufacturer:
https://www.cellsignal.com/products/primary-antibodies/phospho-chk1-ser345-133d3-rabbit-mab/2348
MCM4 (Cell Signaling, #3228S) antibody was validated for WB on human cancer cells by the manufacturer:
https://www.cellsignal.com/products/primary-antibodies/mcm4-antibody/3228
CDC45 (Santa Cruz, #sc-55569) antibody was validated for WB on human cancer cells by the manufacturer:
https://www.scbt.com/p/cdc45-antibody-g-12
SLD5 (Santa Cruz, #sc-398784) antibody was validated for WB on human cancer cells by the manufacturer:
https://www.scbt.com/p/sld5-antibody-d-7
H3 (Santa Cruz, #sc-517576) antibody was validated for WB on human cancer cells by the manufacturer:
https://www.scbt.com/p/histone-h3-antibody-1g1
MCM7 (Santa Cruz, #sc-9966) antibody was validated for WB and IHC on human cancer cells by the manufacturer:
https://www.scbt.com/p/mcm7-antibody-141-2
POLE2 (Santa Cruz, #sc-398582) antibody was validated for WB on human cancer cells by the manufacturer:
https://www.scbt.com/p/dna-pol-epsilon-b-antibody-c-9
PCNA (Santa Cruz, #sc-56) antibody was validated for WB, IF, FACS, and IHC, on human cancer cells by the manufacturer:
https://www.scbt.com/p/pcna-antibody-pc10
FLAG (Sigma, F3165-1MG) antibody was validated for WB on human cancer cells by the manufacturer:
https://www.sigmaaldrich.com/EE/en/product/sigma/f3165
myc (Cell Signaling, #2276) antibody was validated for WB on human cancer cells by the manufacturer:
https://www.cellsignal.com/products/primary-antibodies/myc-tag-9b11-mouse-mab/2276
BrdU Clone B44 (BD, #347580) and CldU (ab6326) antibodies are used routinely for the vast majority of DNA fiber and DNA combing experiments. For example – Vaitsiankova et al, NSMB, 2022; Moore et al., STAR Protocols, 2022.
MCM6 (Abcam ab236151) antibody was validated by the manufacturer for WB, FACS, IF, IHC on human cancer cells:
https://www.abcam.com/mcm6-antibody-epr17686-bsa-and-azide-free-ab236151.html
goat anti-mouse AlexaFluor 488 (Invitrogen, #A-11001) antibody was validated by the manufacturer:
https://www.thermofisher.com/antibody/product/Goat-anti-Mouse-IgG-H-L-Cross-Adsorbed-Secondary-Antibody-Polyclonal/A-11001
goat anti-rat AlexaFluor 594 (Invitrogen, #A-11007) antibody was validated by the manufacturer:
https://www.thermofisher.com/antibody/product/Goat-anti-Rat-IgG-H-L-Cross-Adsorbed-Secondary-Antibody-Polyclonal/A-11007
Goat anti-Mouse Alexa Fluor 488 (Invitrogen # A11029) antibody was validated by the manufacturer:
https://www.thermofisher.com/antibody/product/Goat-anti-Mouse-IgG-H-L-Highly-Cross-Adsorbed-Secondary-Antibody-Polyclonal/A-11029

Eukaryotic cell lines

Policy information about cell lines

Cell line source(s): 293FT from Thermofisher (#R70007), U2OS (HTB-96 from ATCC), POLE1-mAID clones 16 and 1.6, and dcat clones were derived from U2OS cells.

Authentication: we did not authenticate the cell lines

Mycoplasma contamination: all cell lines tested negative for mycoplasma (PCR)

Commonly misidentified lines (See ICLAC register): no commonly misidentified cell lines were used in the study

Flow Cytometry

Plots

Confirm that:

☒ The axis labels state the marker and fluorochrome used (e.g. CD4-FITC).

☒ The axis scales are clearly visible. Include numbers along axes only for bottom left plot of group (a 'group' is an analysis of identical markers).

☒ All plots are contour plots with outliers or pseudocolor plots.

☒ A numerical value for number of cells or percentage (with statistics) is provided.

Methodology

Sample preparation: For EdU FACS, cells were treated with 10 µM EdU for 10 min, trypsinized, washed with PBS, and fixed with cold 70% ethanol on ice for 30 min to overnight. Cells were washed with PBS, and EdU staining was performed by using the EdU Click-iT kit (Thermofisher, # C10632), according to the manufacturer's instructions. For DNA staining, we used 7-AAD (7-Aminoactinomycin D) (Thermofisher, # A1310) or FxCycle™ PI/RNase Staining Solution (Thermofisher, #F10797).
Chromatin association of MCM was assessed essentially as described (45), except anti-MCM7 antibody (Santa Cruz, #sc-9966) at 1:200 dilution was used for immunostaining, and 7-AAD (7-Aminoactinomycin D) (Thermofisher, # A1310) was used for DNA staining.

For the experiments studying the chromatin association of MCM, after trypsinization and PBS wash, cells were extracted with CSK buffer, fixed with 4% paraformaldehyde, and blocked with 5% BSA followed by immunostaining with anti-MCM7 antibody (Santa Cruz, #sc-9966) at 1:200 dilution. 7-AAD (7-Aminoactinomycin D) (Thermofisher, # A1310) was used for DNA

staining.

Instrument	BD FACSCalibur
Software	CellQuestPro was used for datacollection, FASCalyzer was used for analysis, statistics and plots were performed in GraphPad Prism 9
Cell population abundance	EdU+ and G1 cell population abundance was quantified based on 4 experimental repeats and the quantification is shown on separate graphs (Fig. 1e, f)
Gating strategy	Supplementary figure 1o shows the standard FSC/SSC gating; Supplementary figure 1j shows G1 and S-phase population gating; supplementary figure 5 shows gating based on the level of EdU incorporation.

☒ Tick this box to confirm that a figure exemplifying the gating strategy is provided in the Supplementary Information.
