# Supplementary material for: The non-catalytic role of DNA polymerase epsilon in replication initiation in human cells
Source: Nat Commun. 2022 Nov 19;13:7099. doi: 10.1038/s41467-022-34911-4 (PMC9675812; doi:10.1038/s41467-022-34911-4)

Figure 1a

POLE1

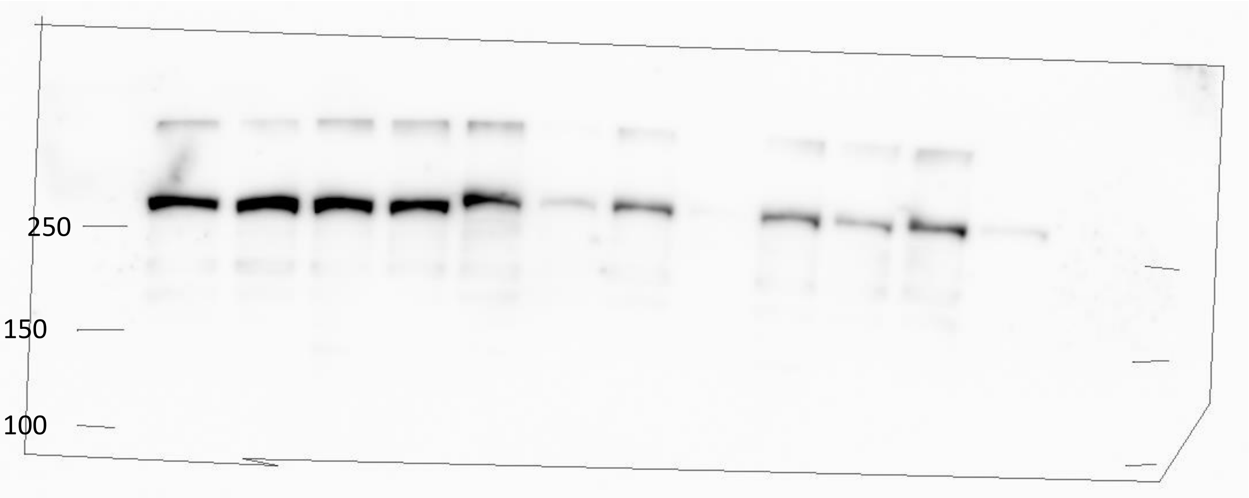

osTIR1

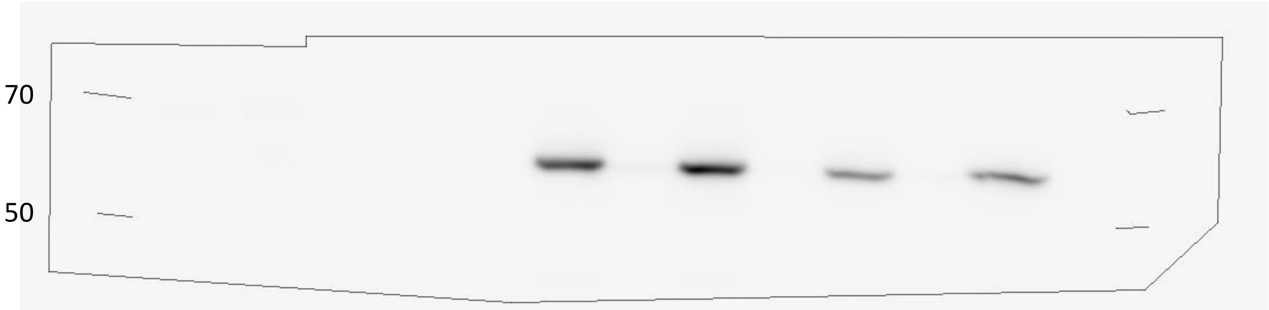

GAPDH

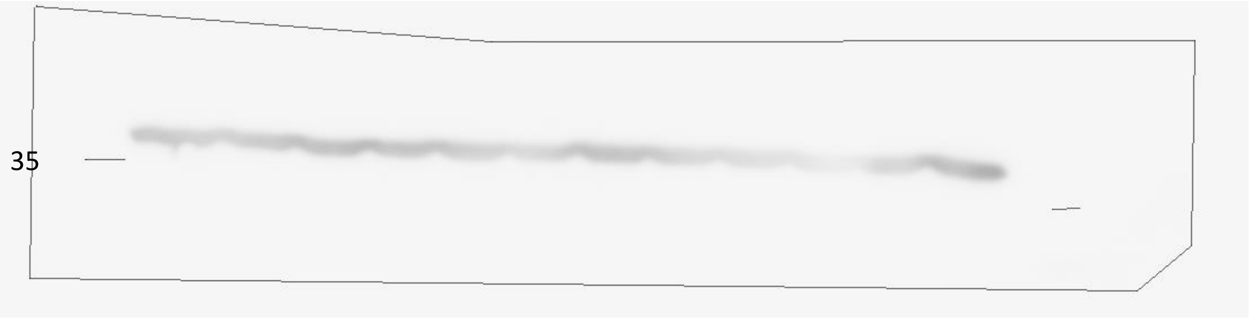

Figure 1c

POLE1

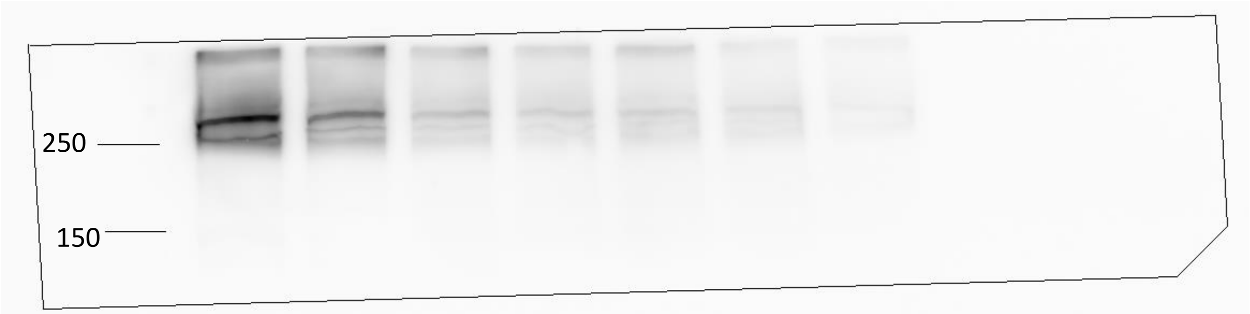

osTIR1

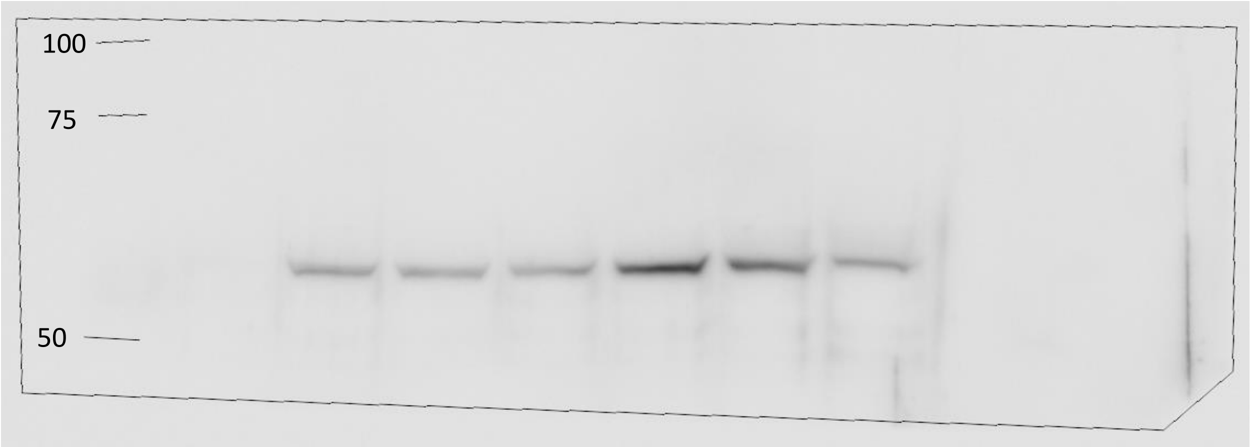

GAPDH

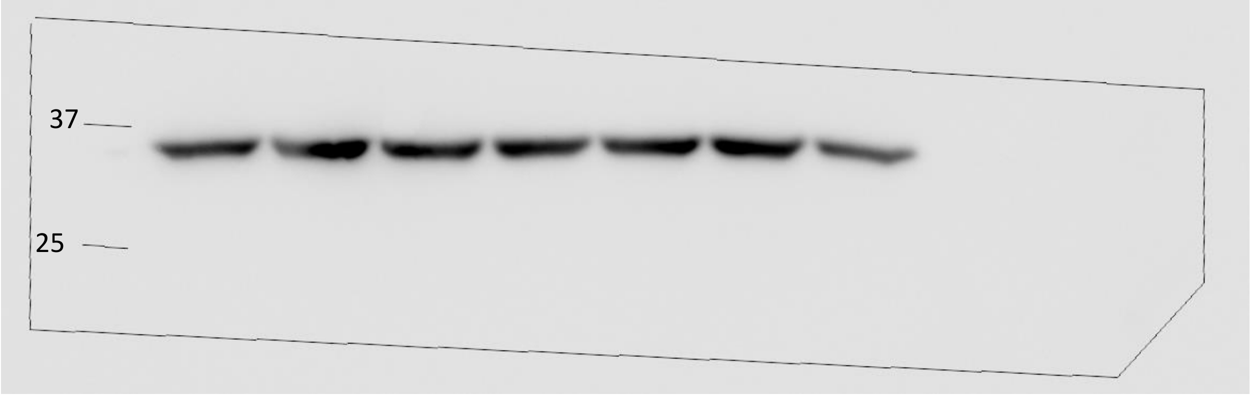

Supplementary figure 1b, DNA gels:

KI:

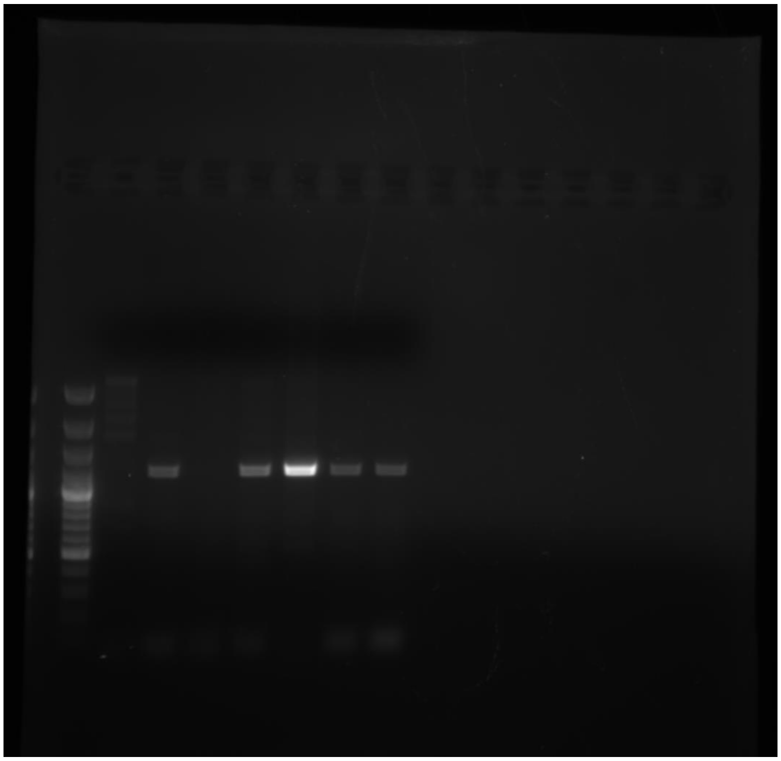

Endogenous:

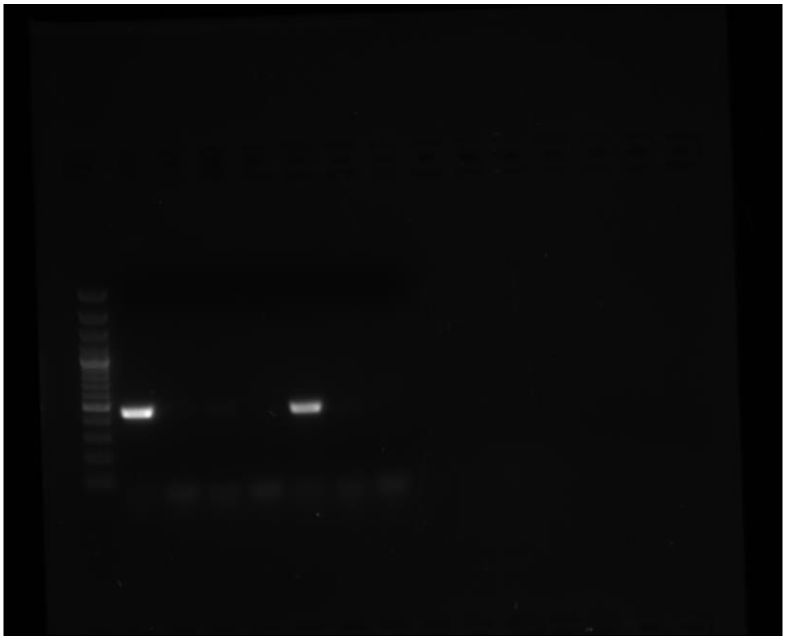

Supplementary figure 1f

POLE1

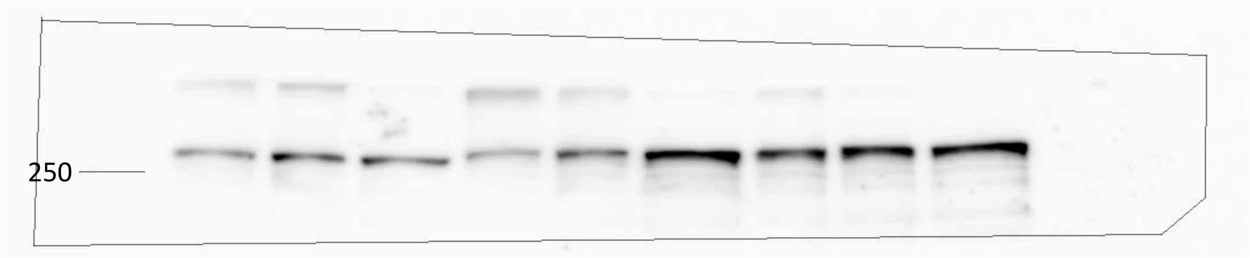

PCNA

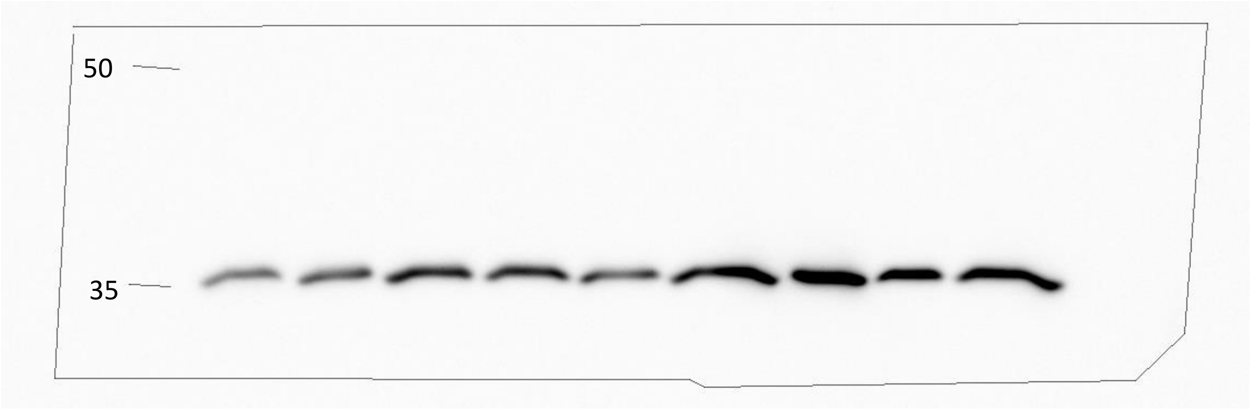

Supplementary figure 1I

POLE1

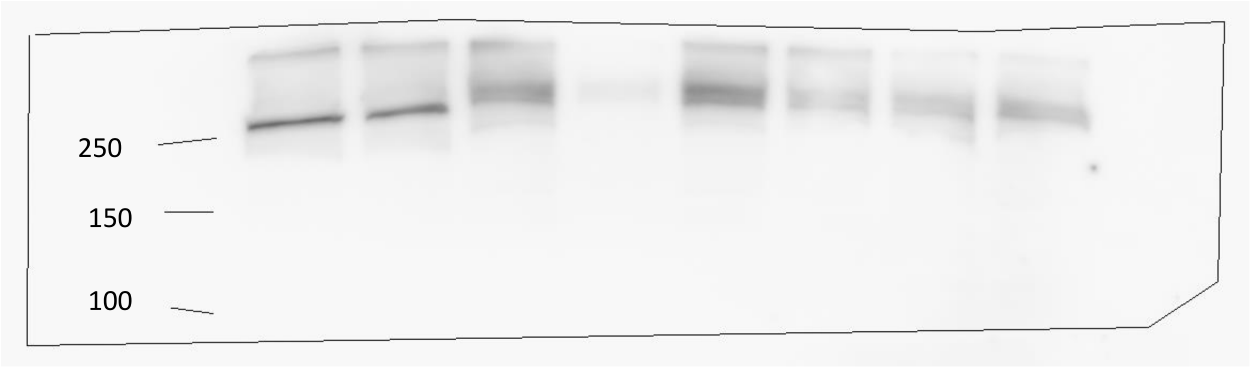

osTIR1

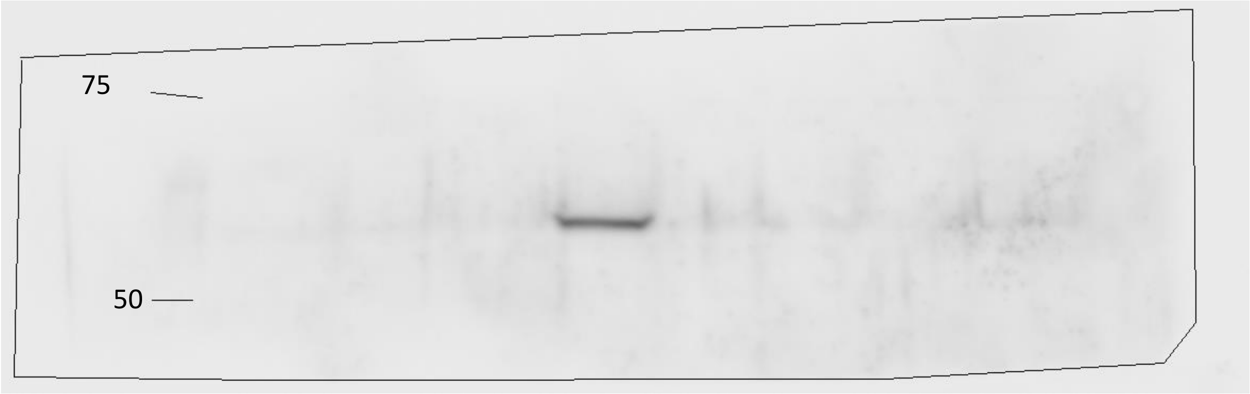

GAPDH

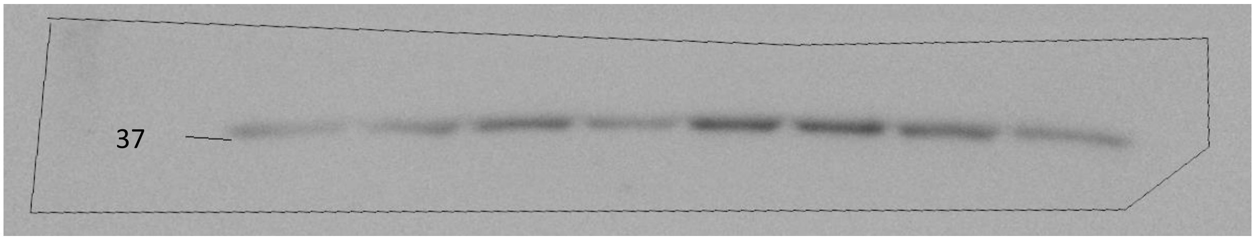

Supplementary figure 1m (only the first four lanes of the blot are shown in the figure)

POLE1

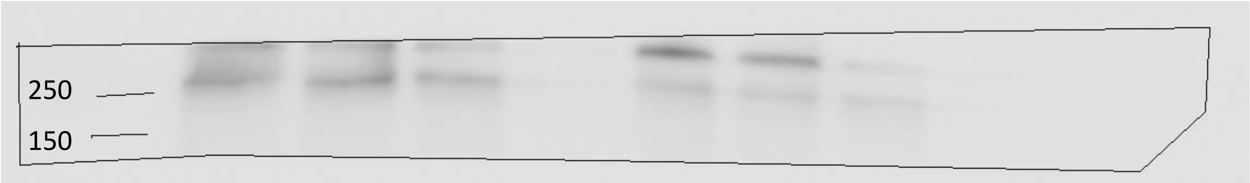

osTIR1

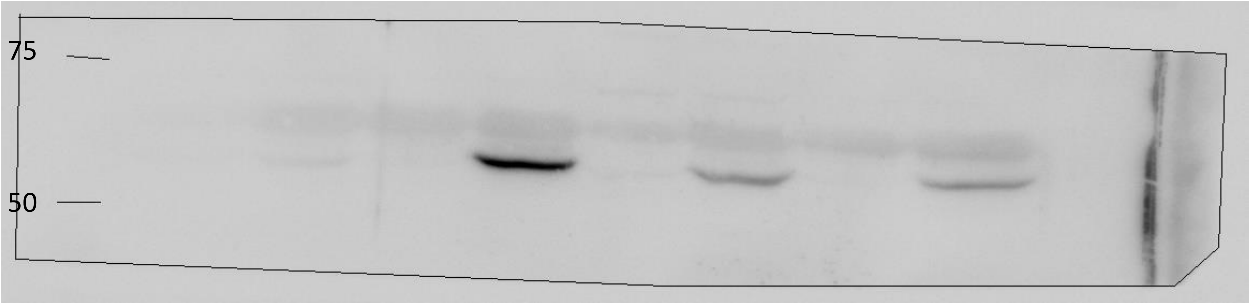

pChk1

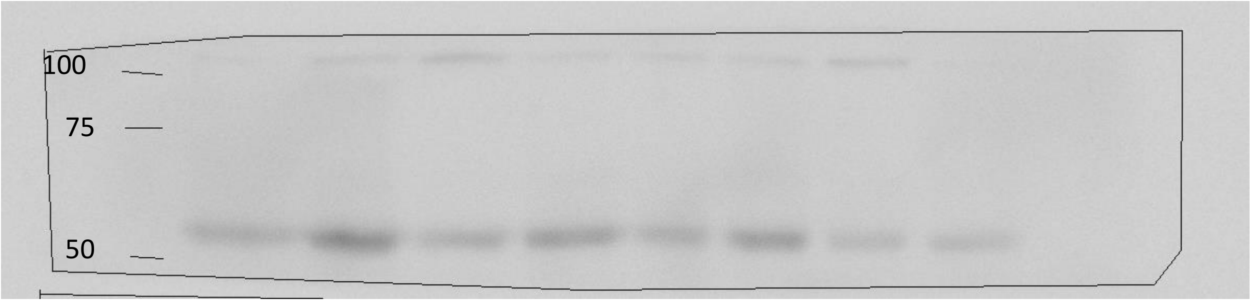

GAPDH

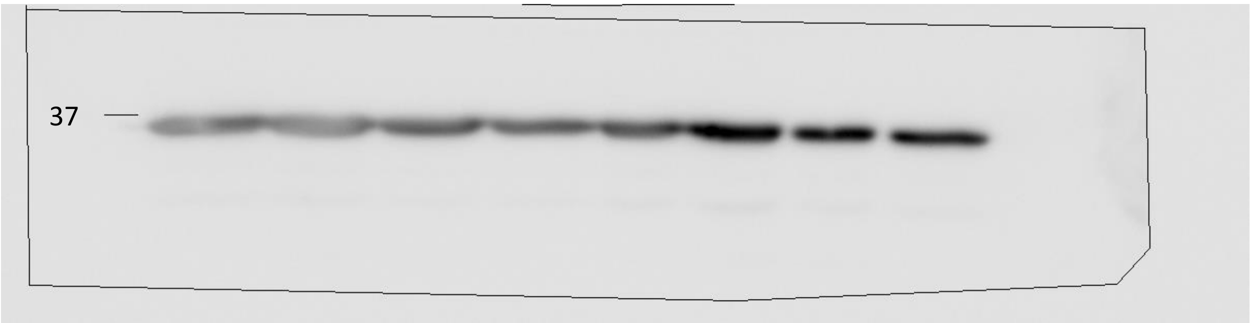

Figure 2a

POLE1

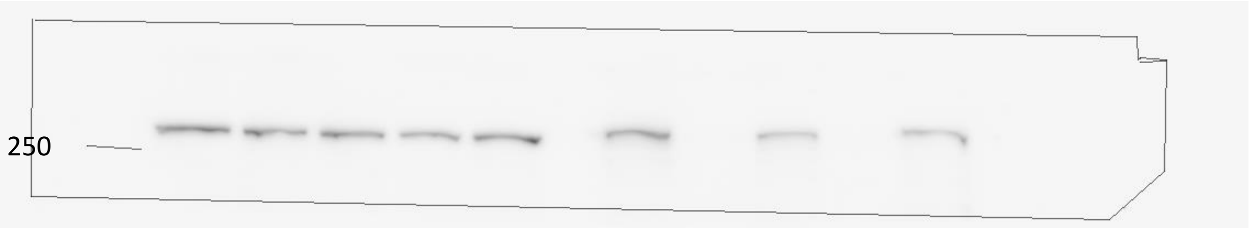

MCM4

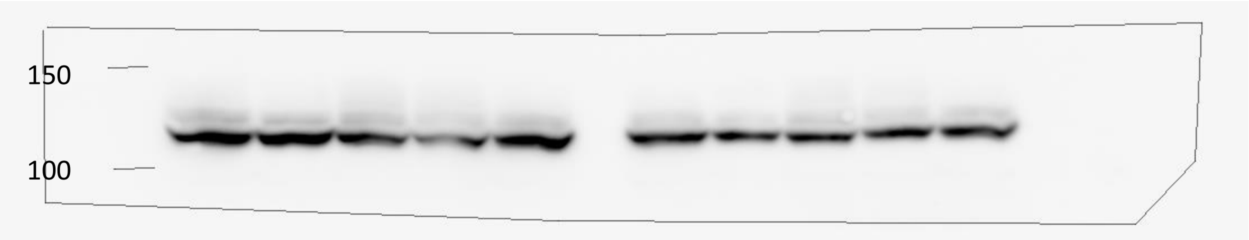

CDC45

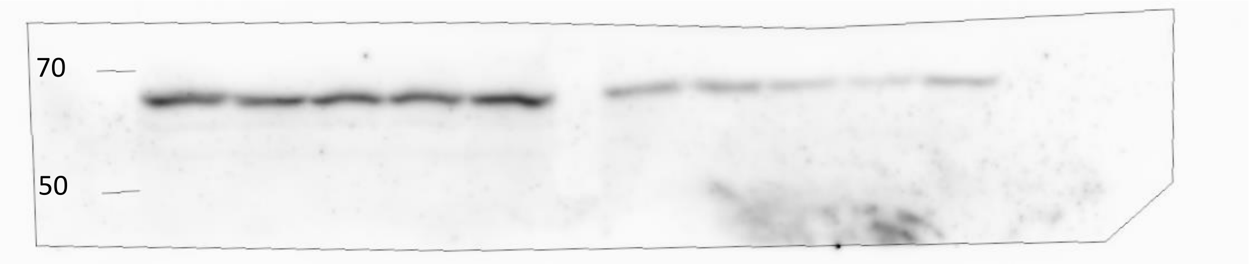

SLD5

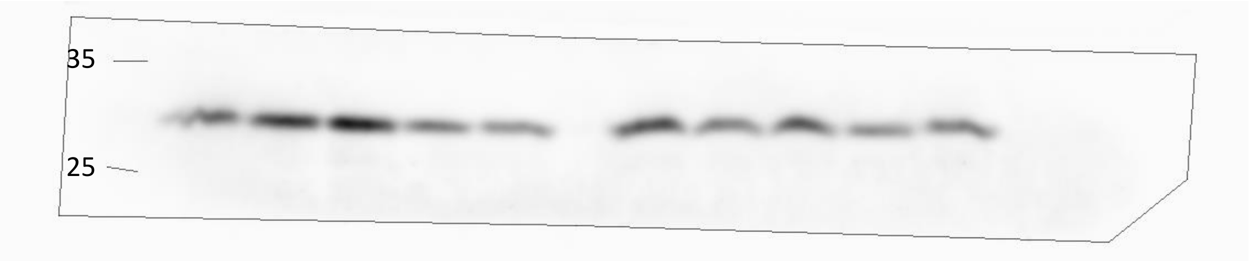

Figure 2a (continued)

pCHK1

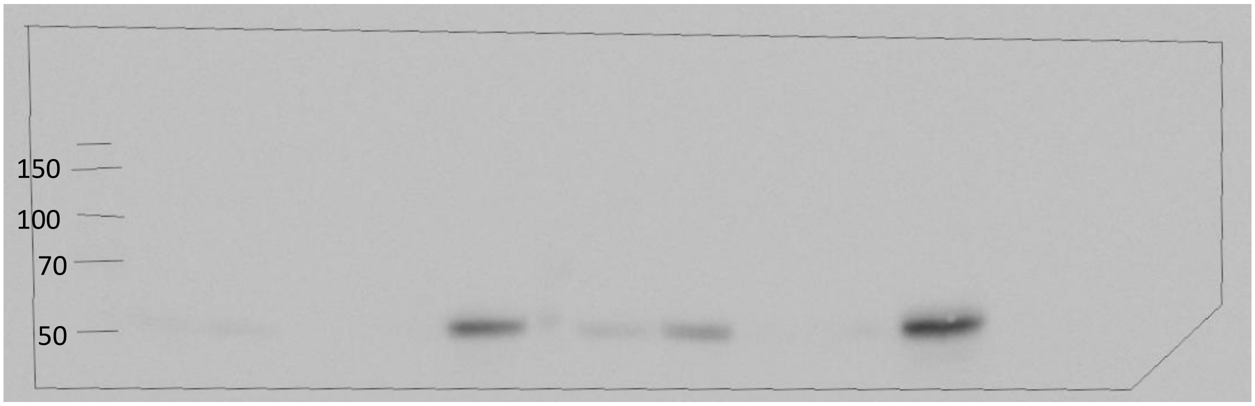

CHK1

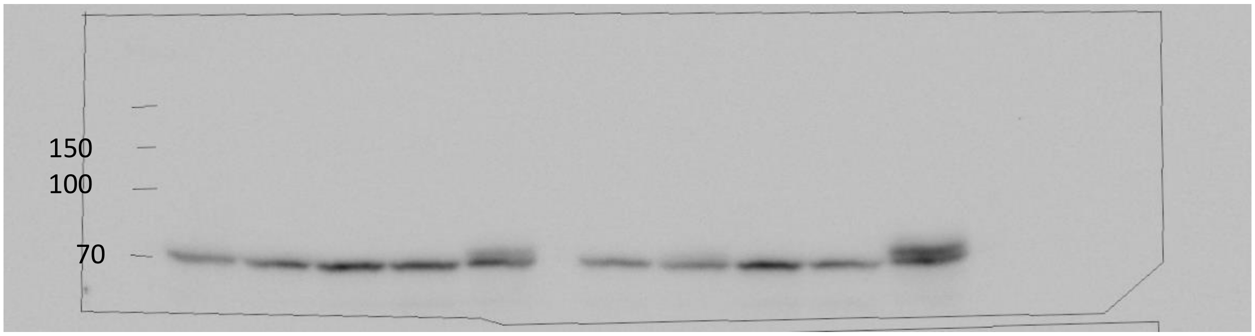

GAPDH

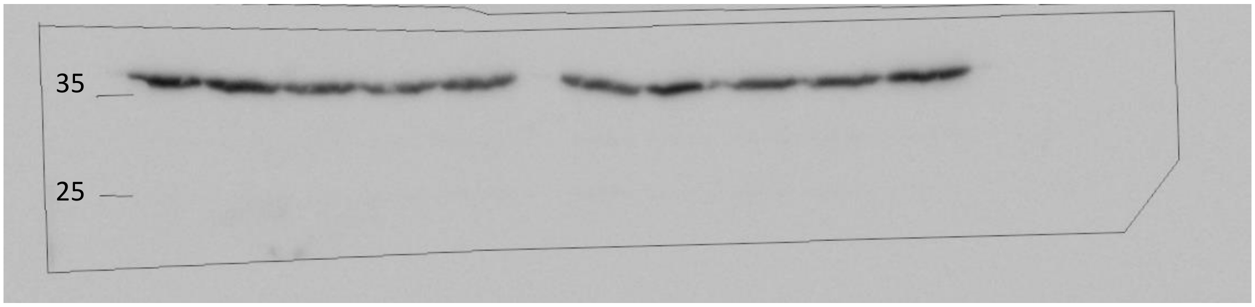

Figure 2b

POLE1

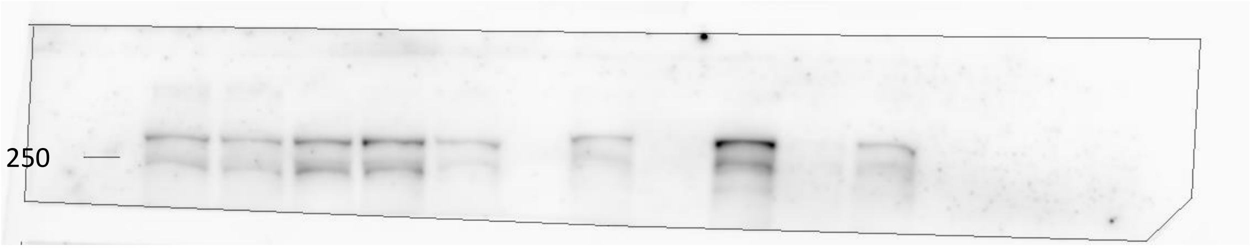

MCM4

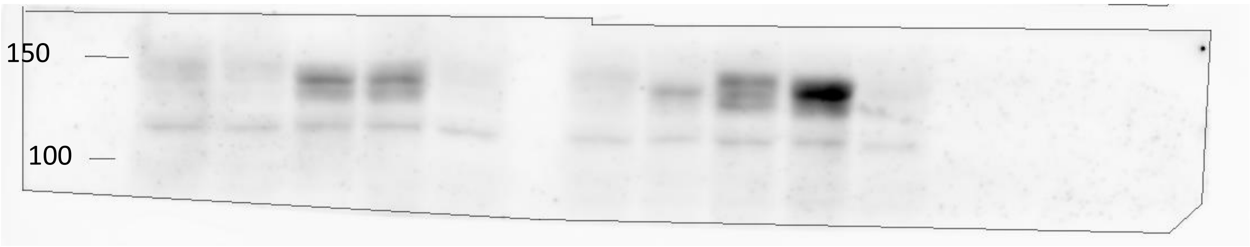

CDC45

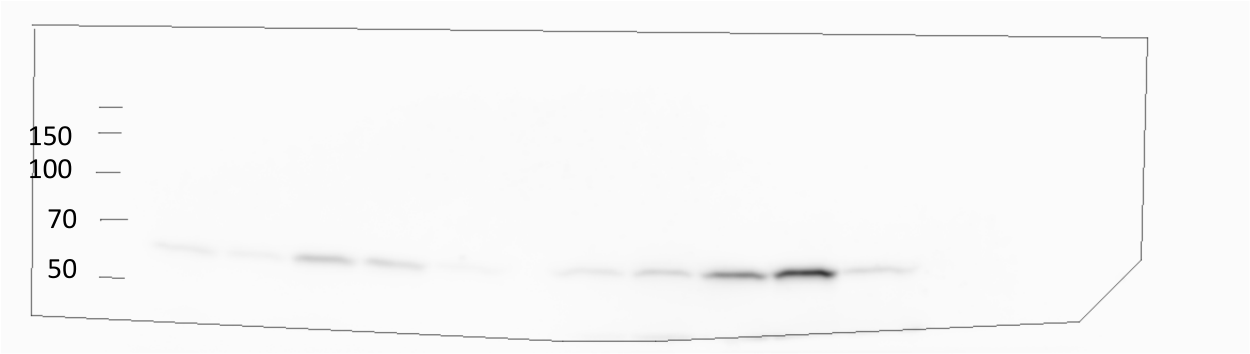

SLD5

*the line above the membrane's upper border is the lower edge of the CDC45 membrane shown above

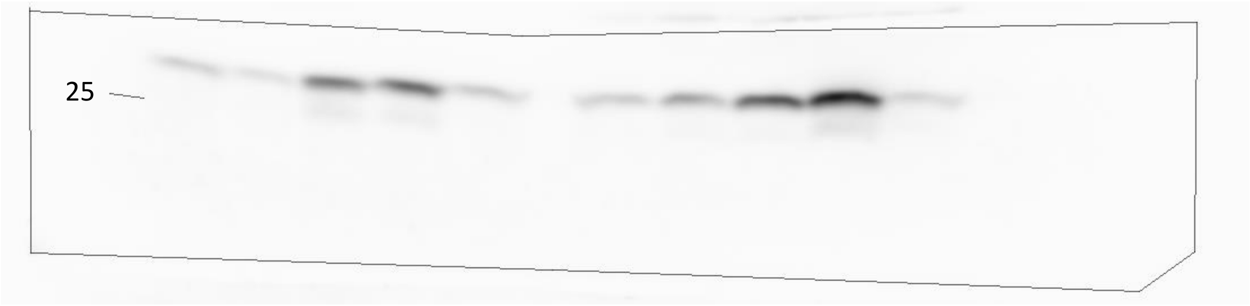

Figure 2b (continued)

Histone H3

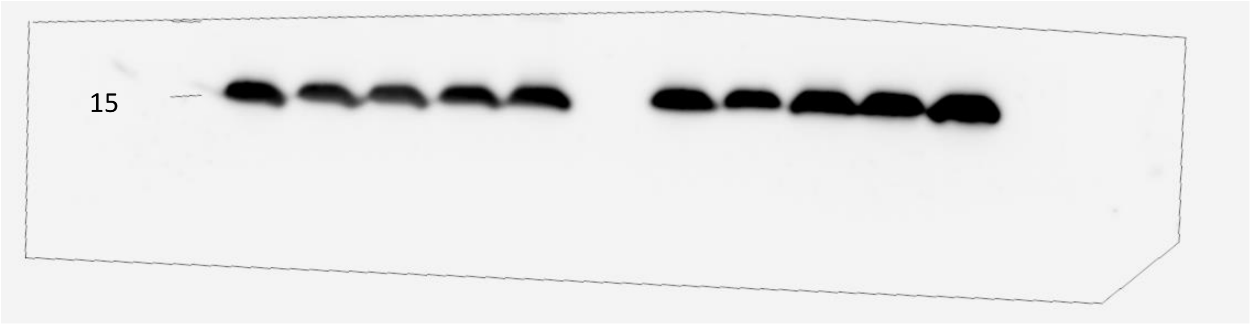

Figure 2c.

POLE1

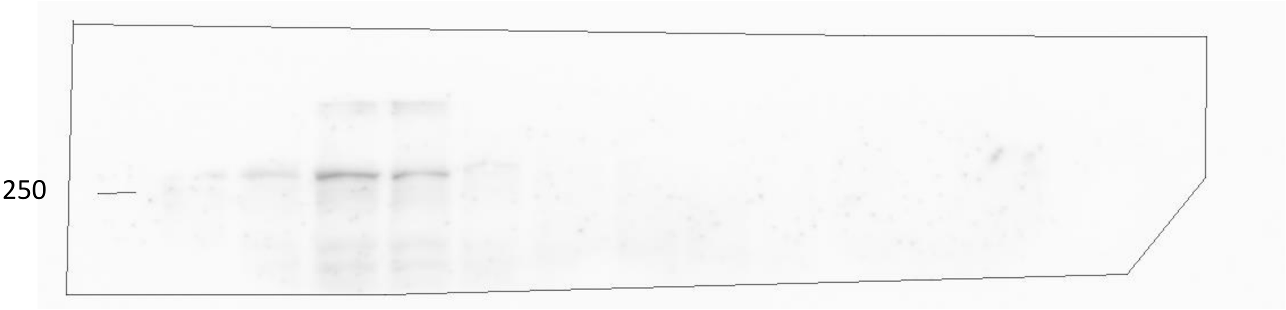

MCM4

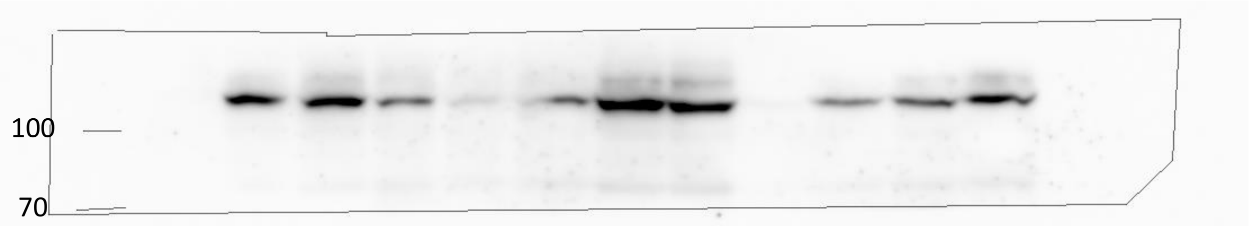

CDC45

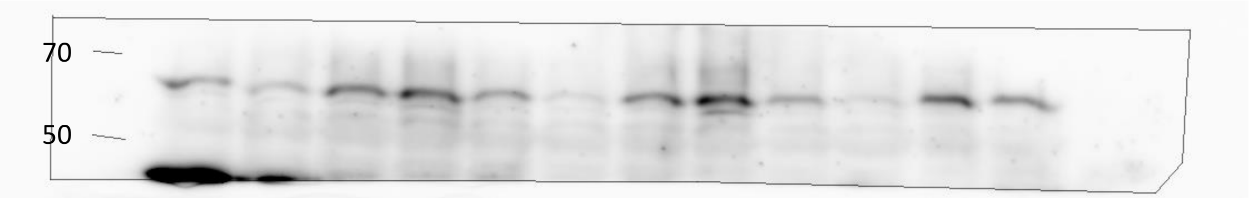

SLD5

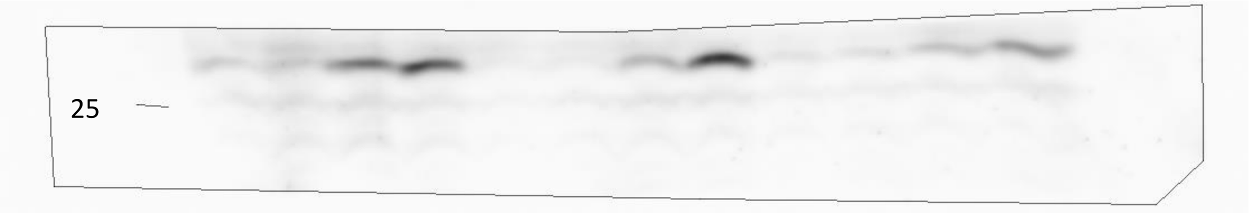

H3

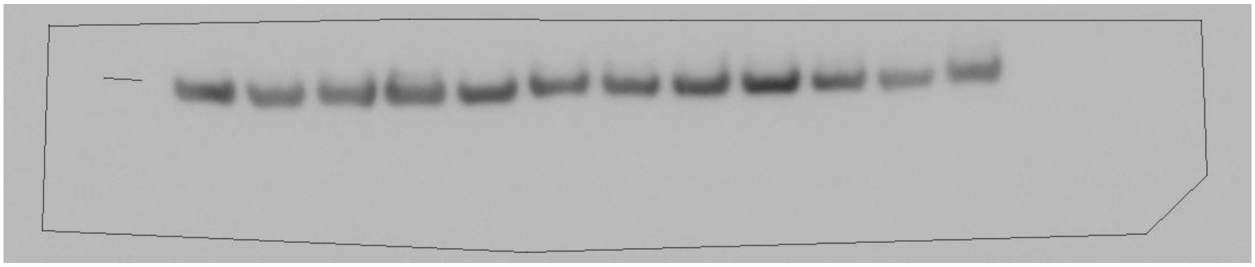

Supplementary figure 2a-b (clone 1.6) First four lanes – NIC, second group of four – soluble.

POLE1

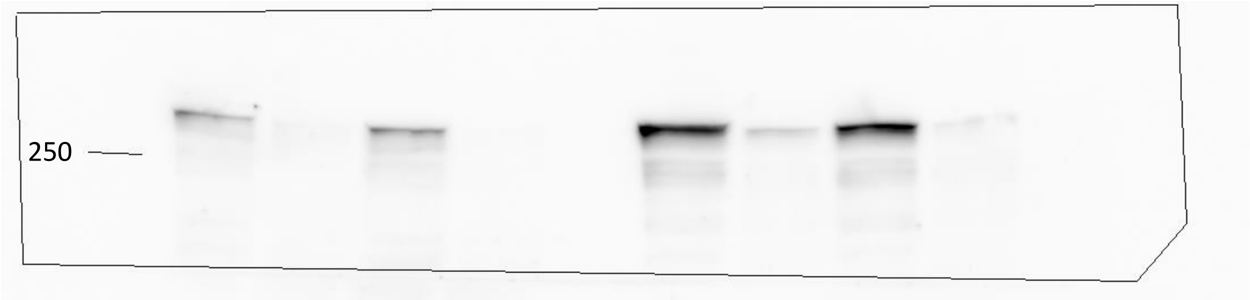

CDC45 (the lower bright band is non-specific, it is below the 50kDa marker band)

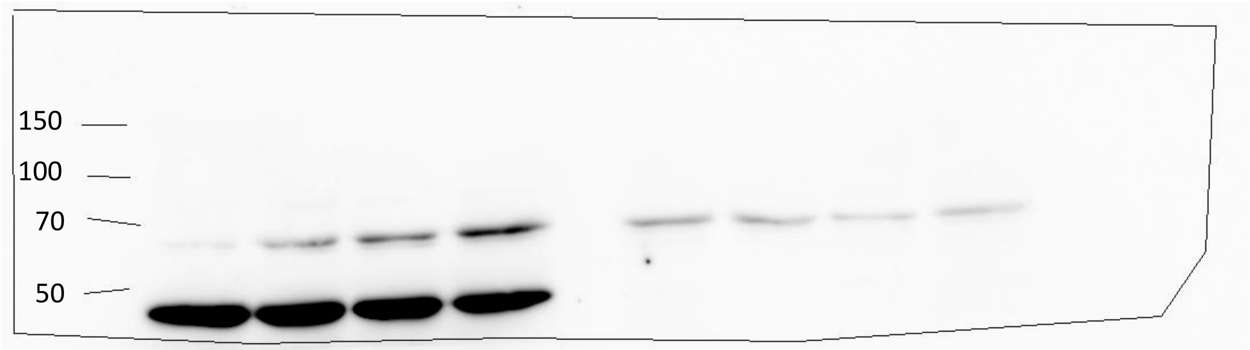

MCM4

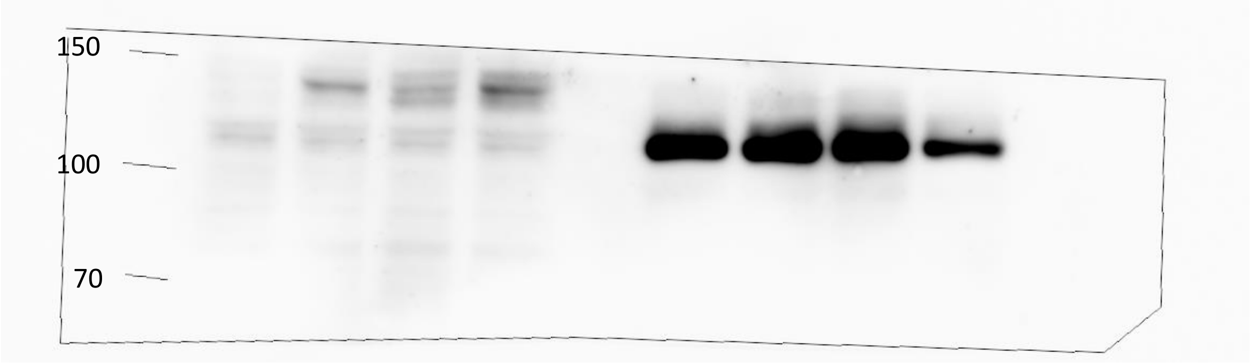

SLD5

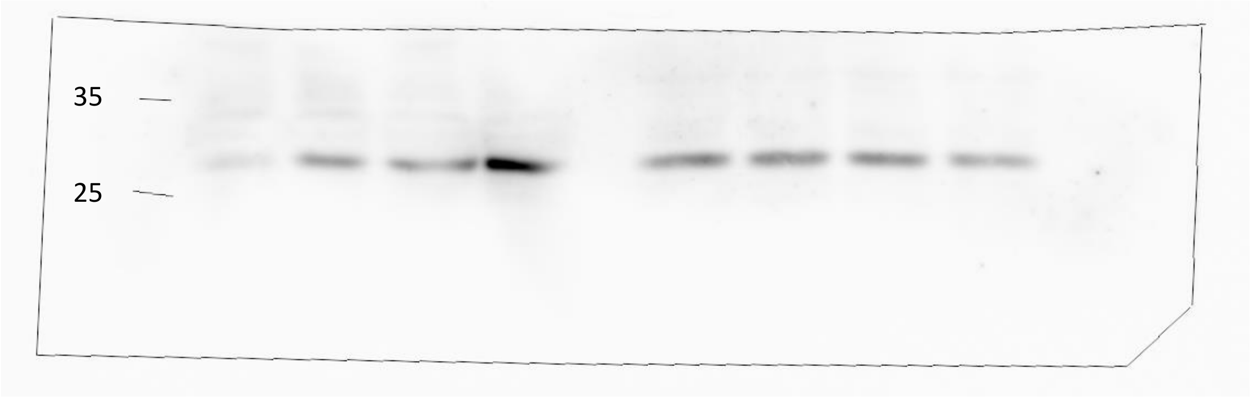

Supplementary figure 2a-b - continued

Histone H3 (NIC – the first four lanes)

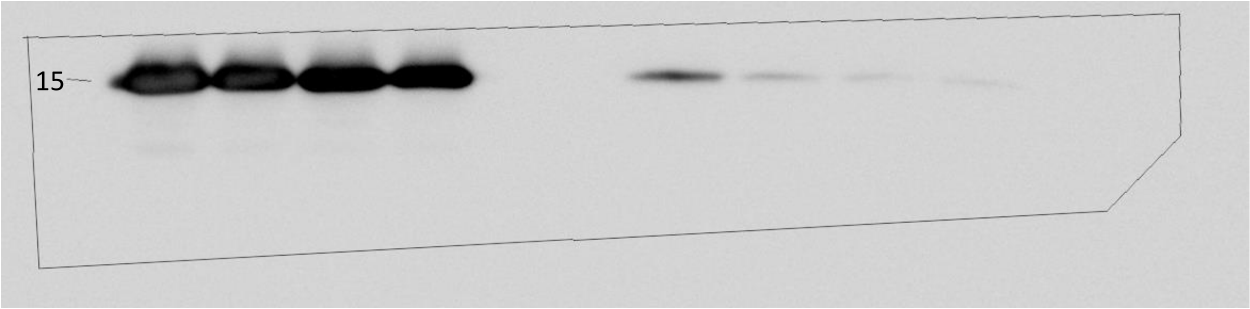

Soluble only (the same four samples were loaded twice for GAPDH, first four lanes were included in the figure):

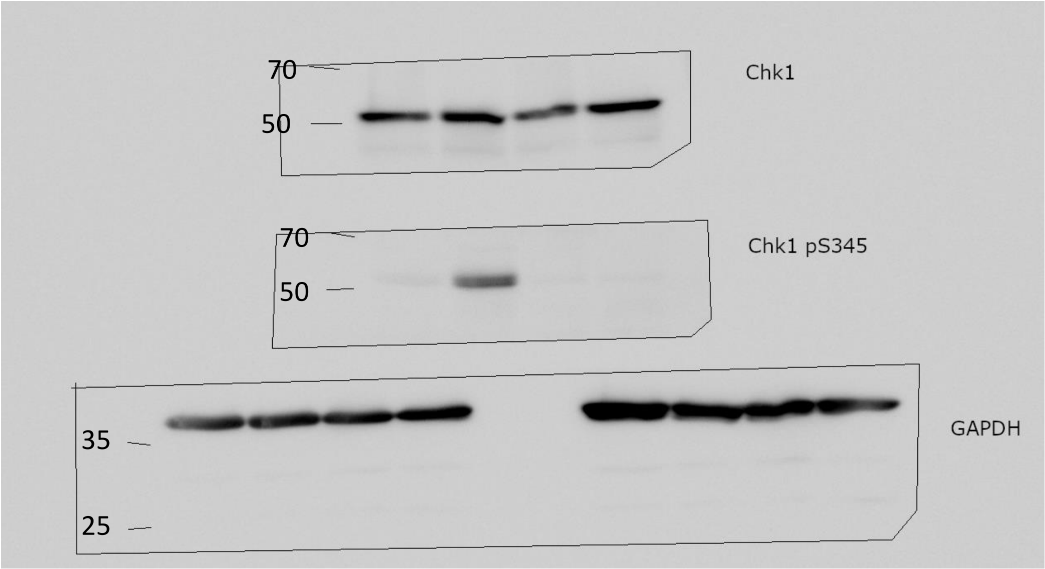

Supplementary Figure 2c.

POLE1

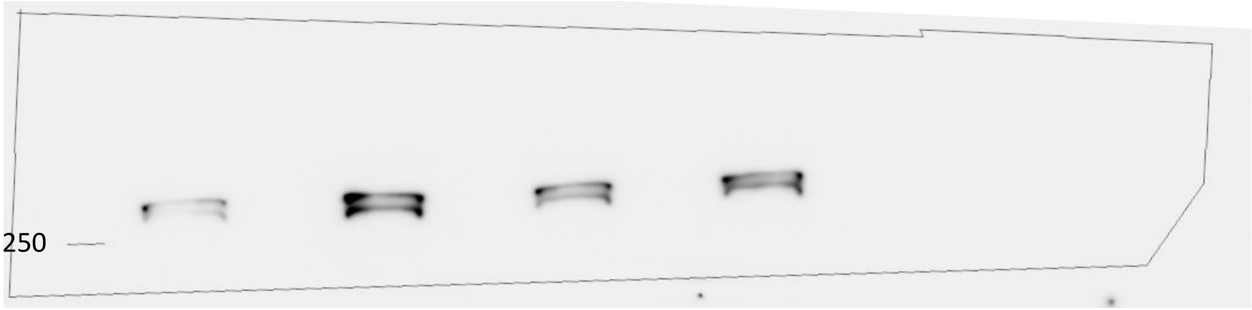

MCM4

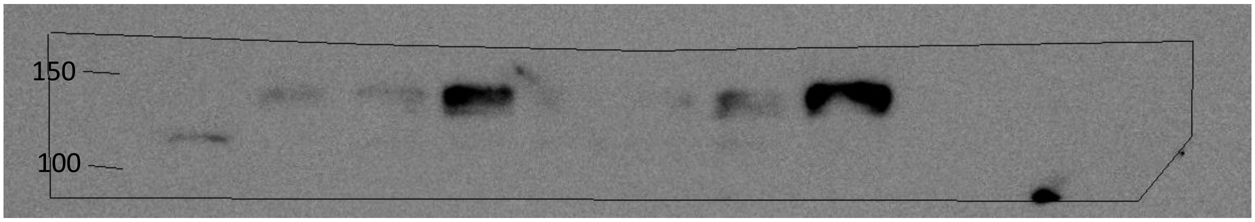

CDC45 (the lower band at the edge is non-specific)

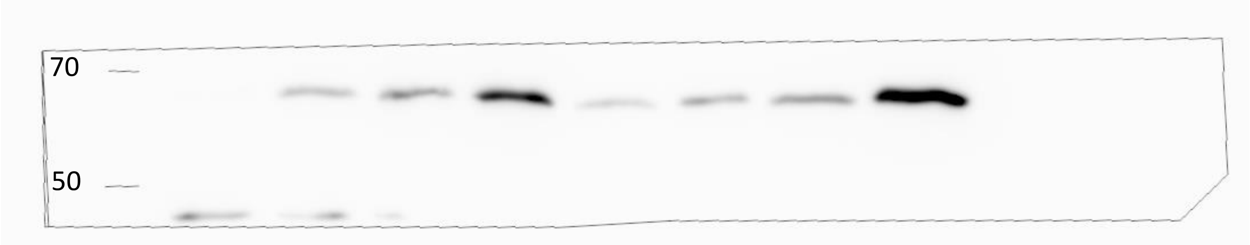

SLD5

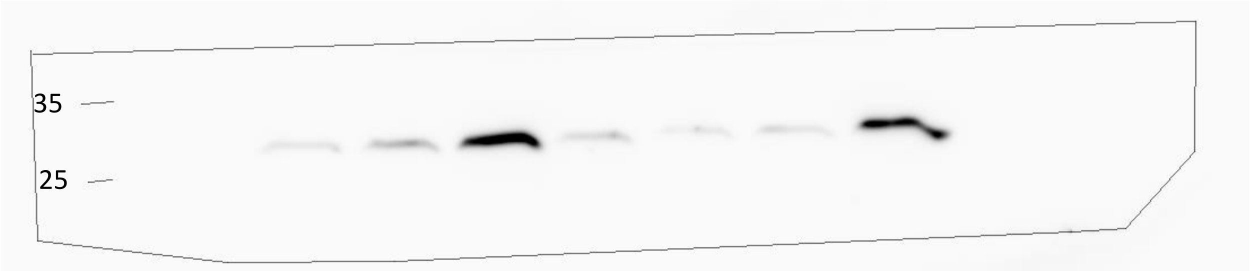

H3

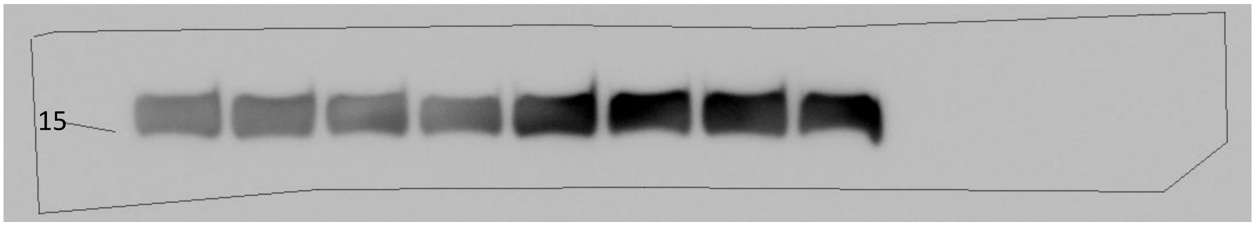

Supplementary Figure 2c (continued).

POLE1 (soluble)

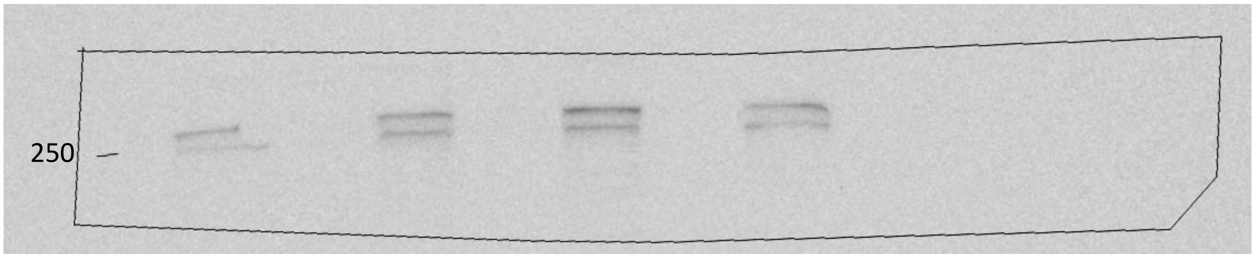

pCHK1 (soluble)

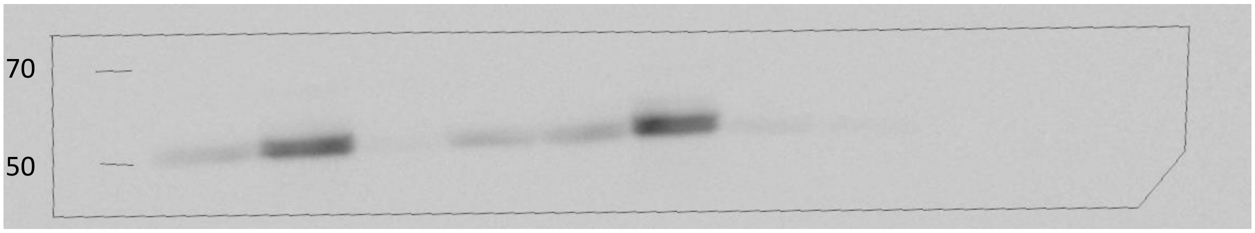

GAPDH (soluble)

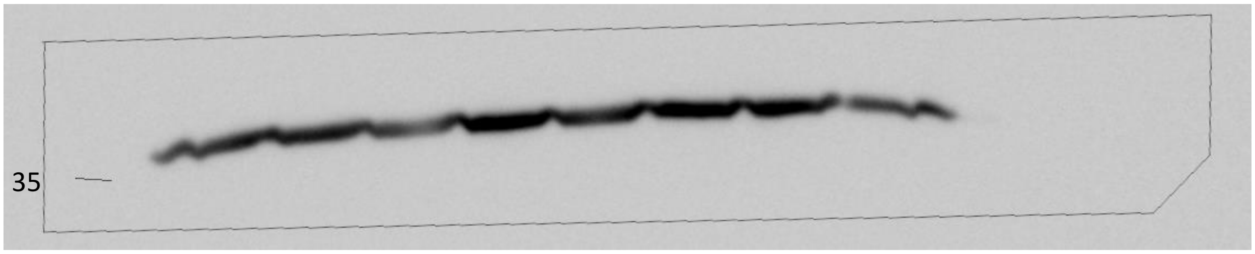

Supplementary Figure 2d.

POLE1

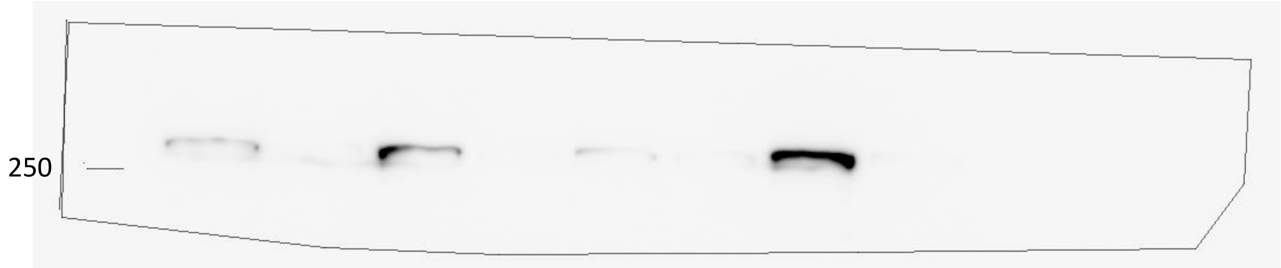

MCM4

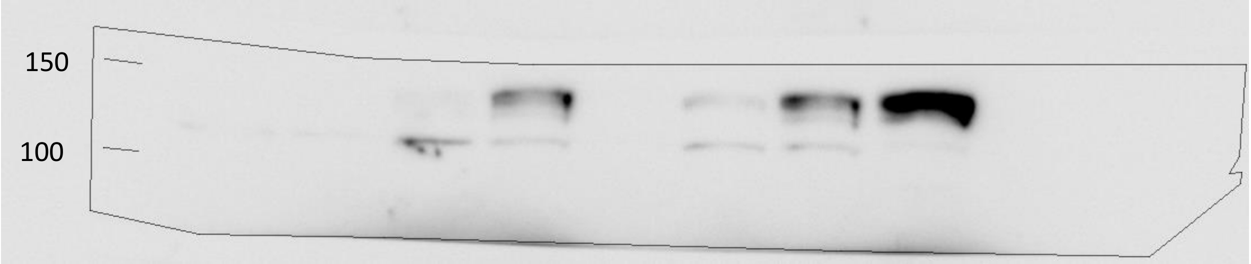

CDC45 (the line below is the upper edge of the SLD5 membrane, the lower band at the edge is non-specific)

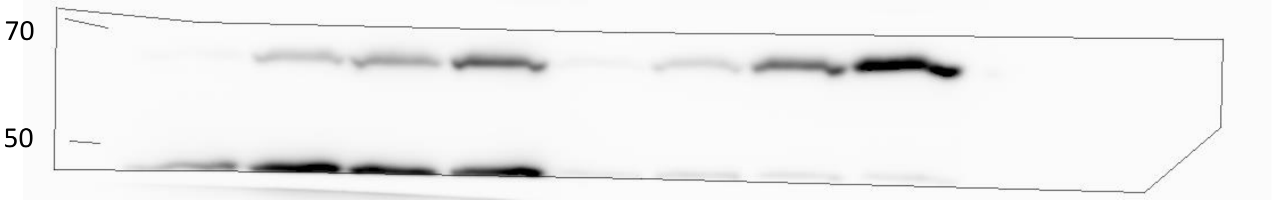

SLD5 (the line above is the lower edge of the CDC45 membrane)

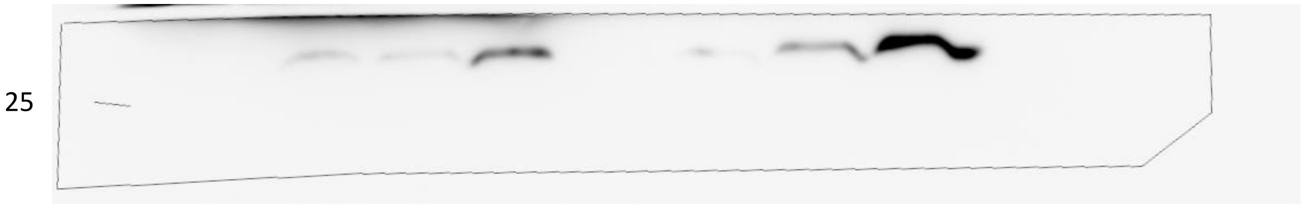

H3

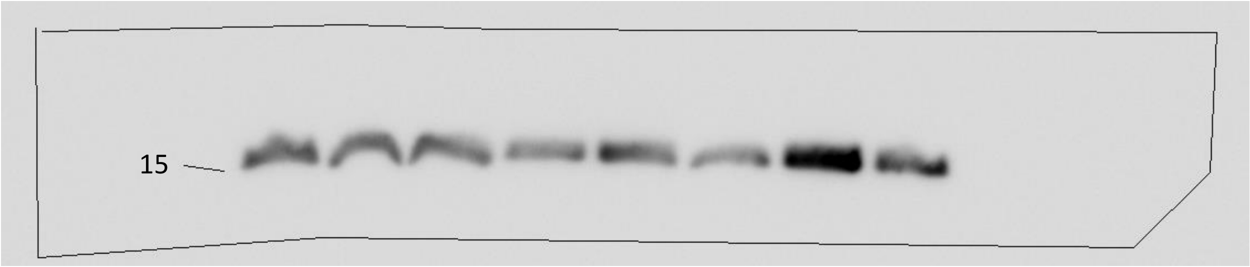

Supplementary Figure 2d (continued).

POLE1 soluble

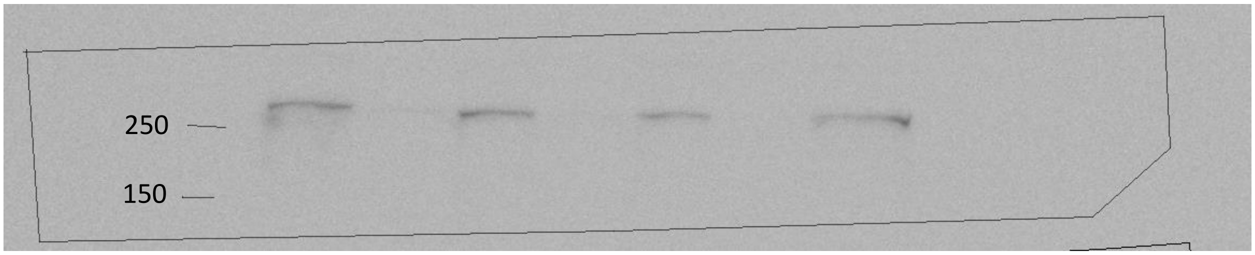

pCHK1 soluble

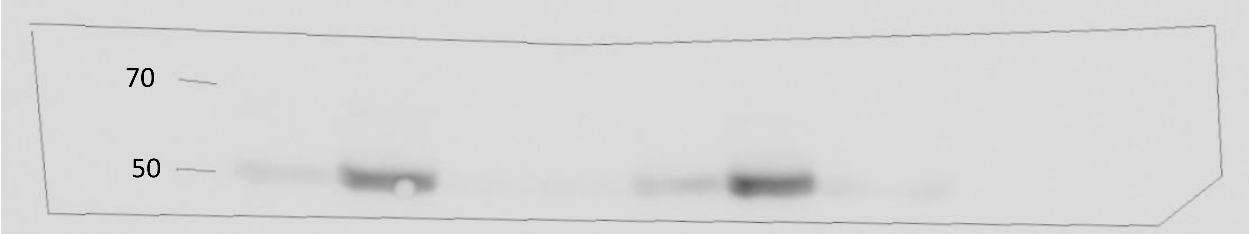

GAPDH soluble

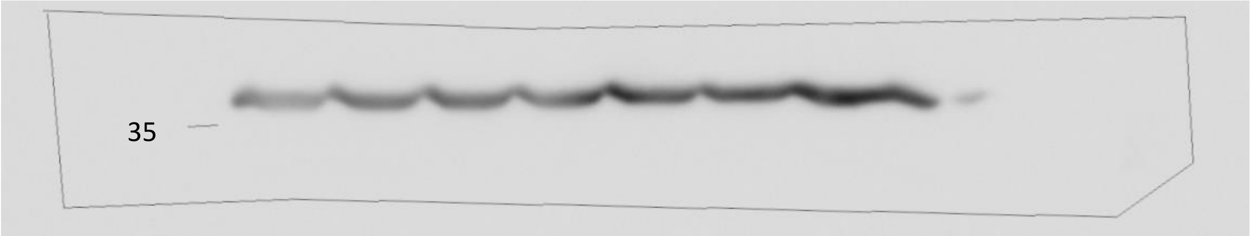

Supplementary Figure 2f.

POLE1

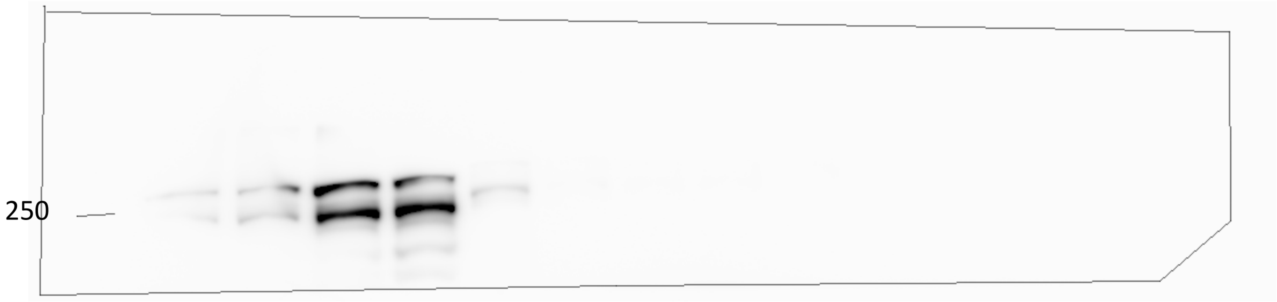

MCM4

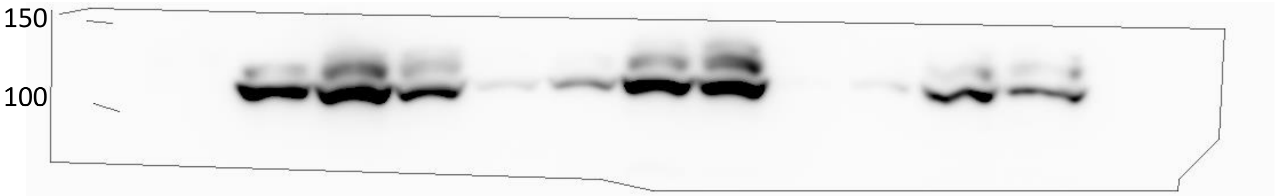

CDC45

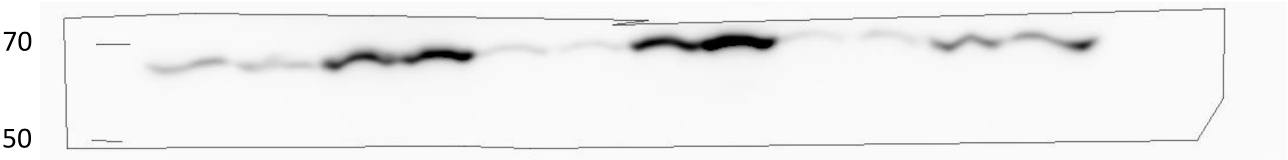

SLD5

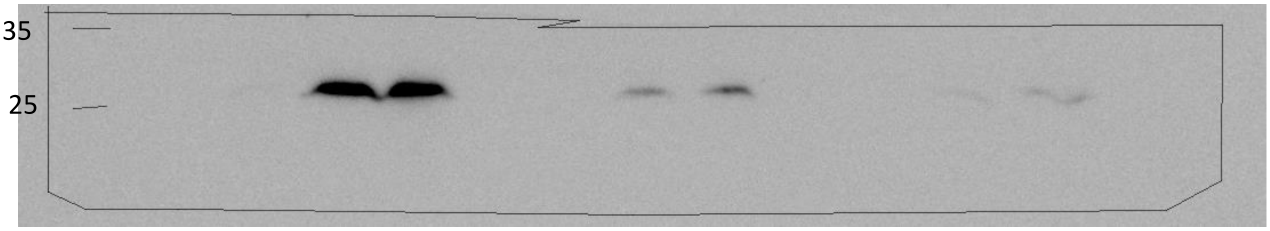

H3

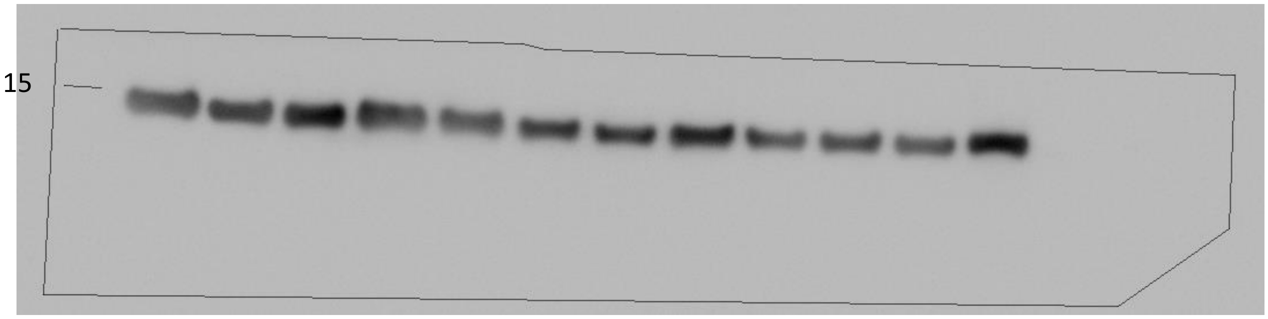

Supplementary Figure 2g.

POLE1

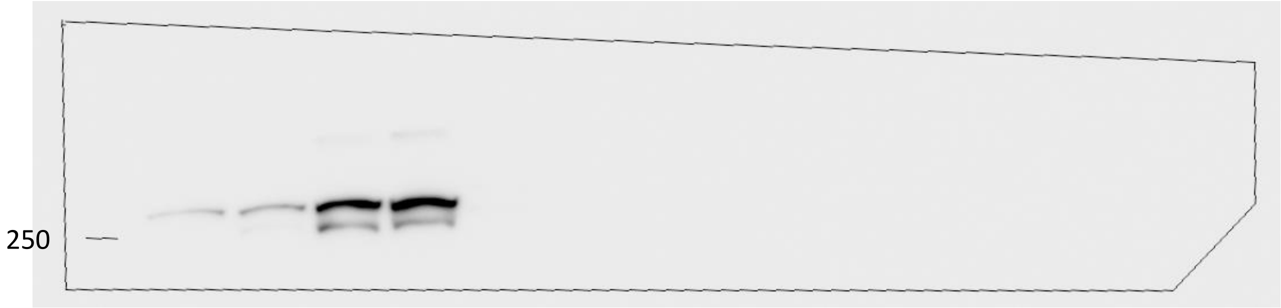

MCM4

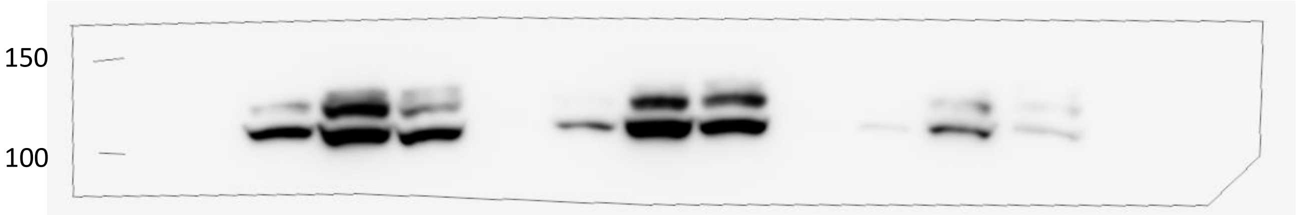

CDC45

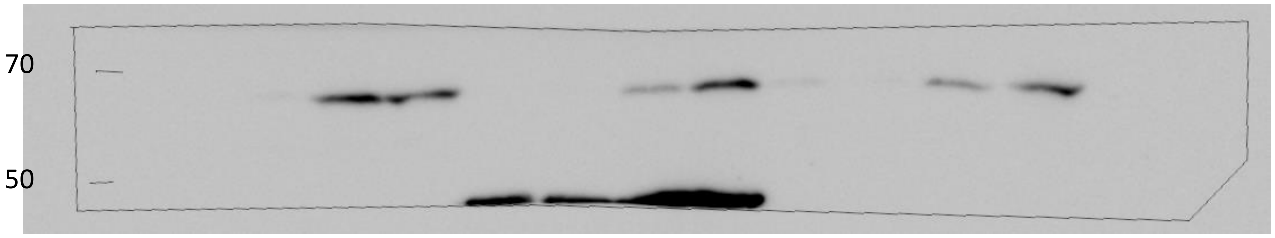

SLD5

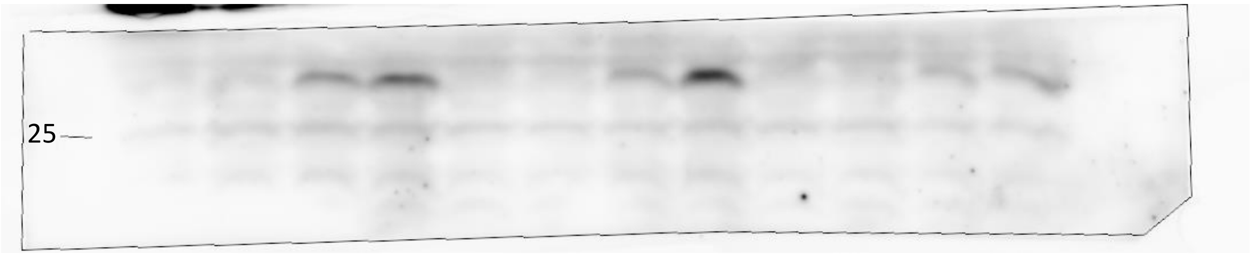

H3

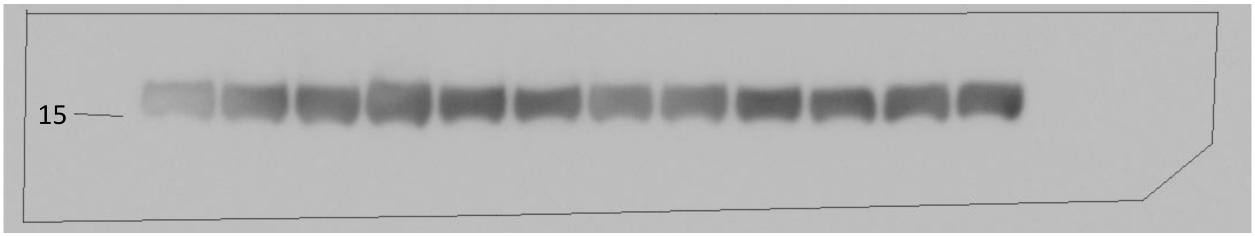

Supplementary Figure 2h.

POLE1

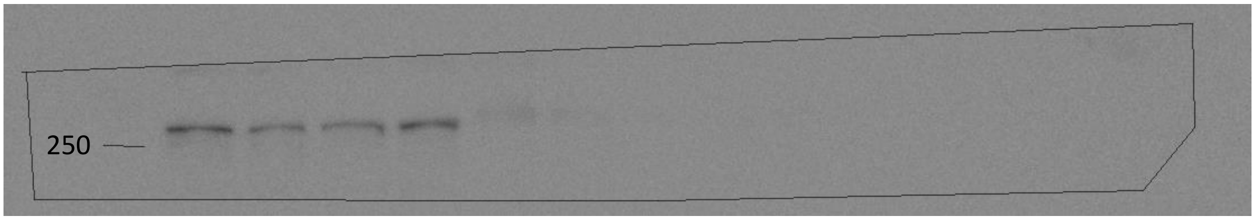

MCM4

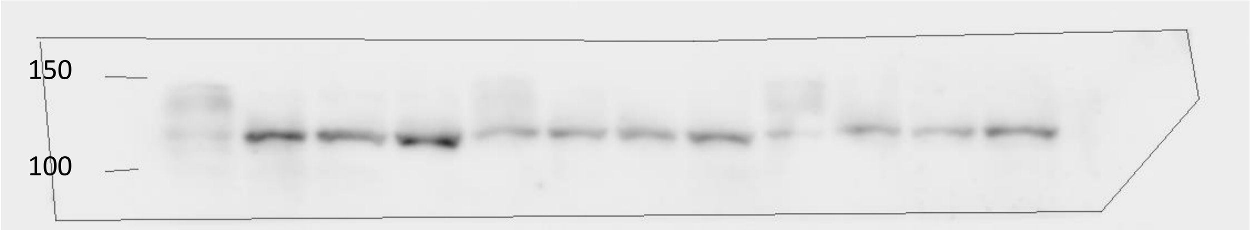

CDC45

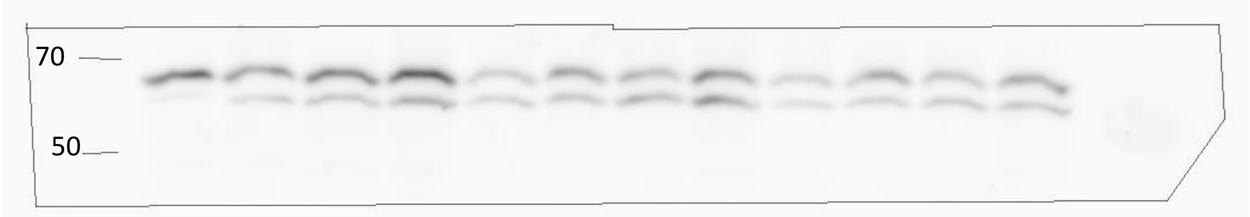

SLD5

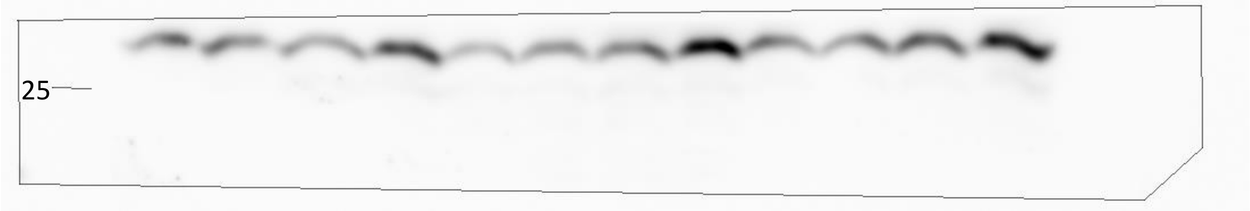

GAPDH

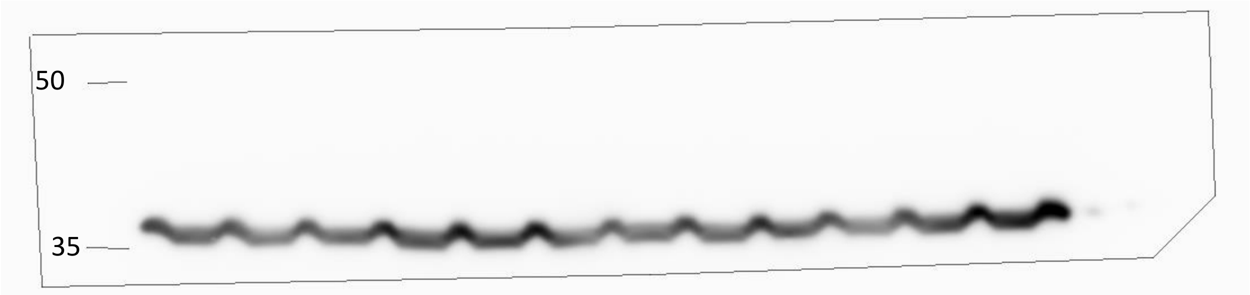

Figure 5b

FLAG

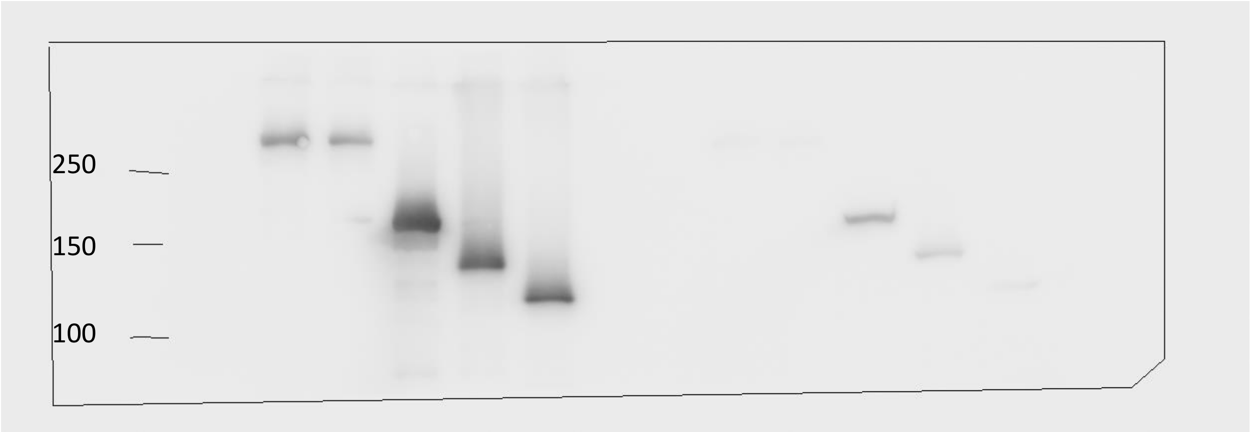

POLE2

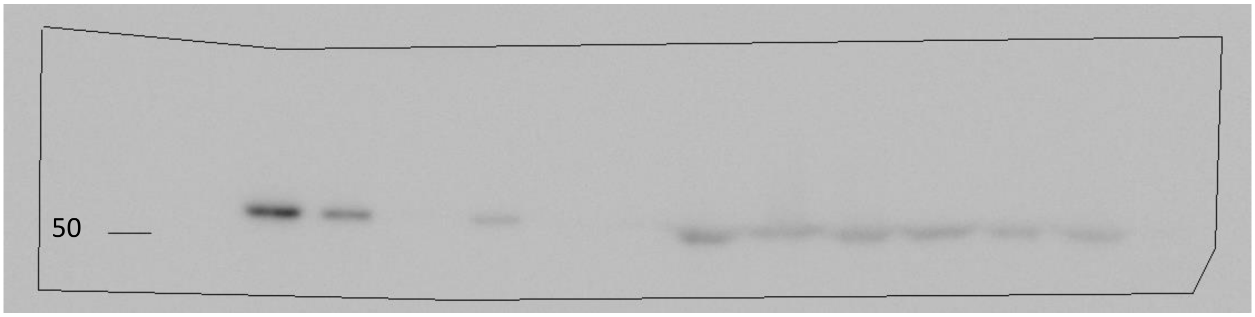

MCM7 (long exposure for IP samples)

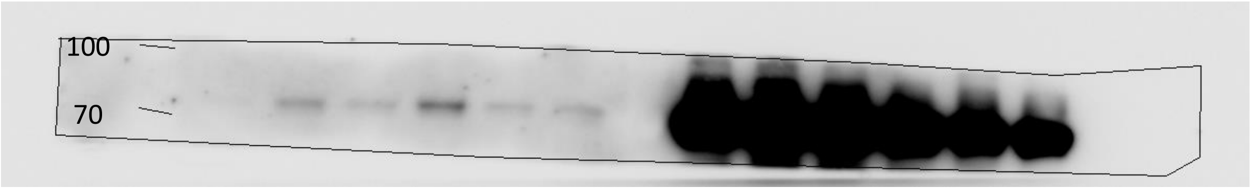

MCM7 (short exposure for input samples)

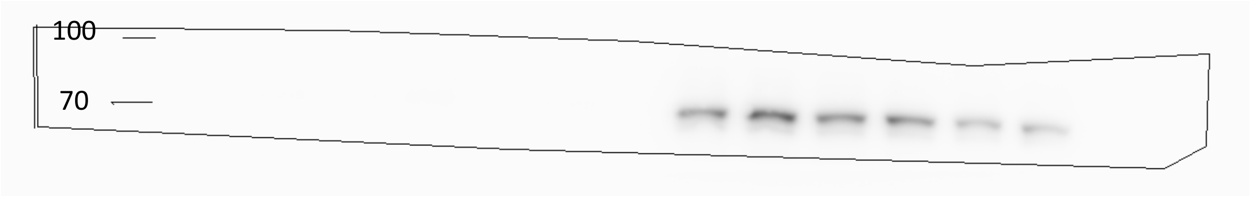

Figure 6a

POLE1

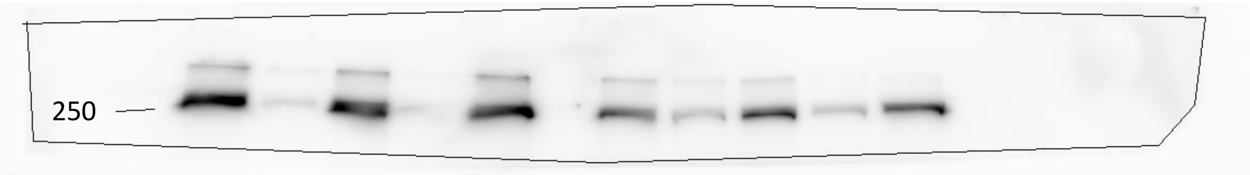

Myc

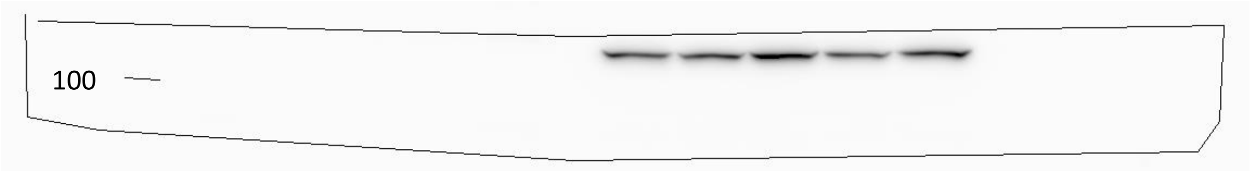

POLE2

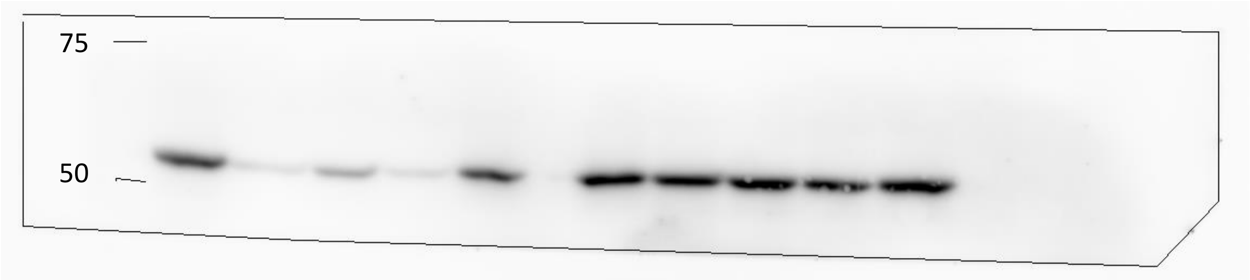

pCHK1

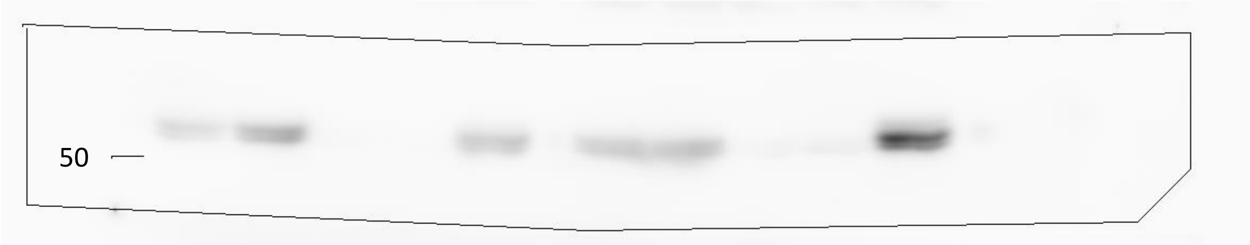

CHK1

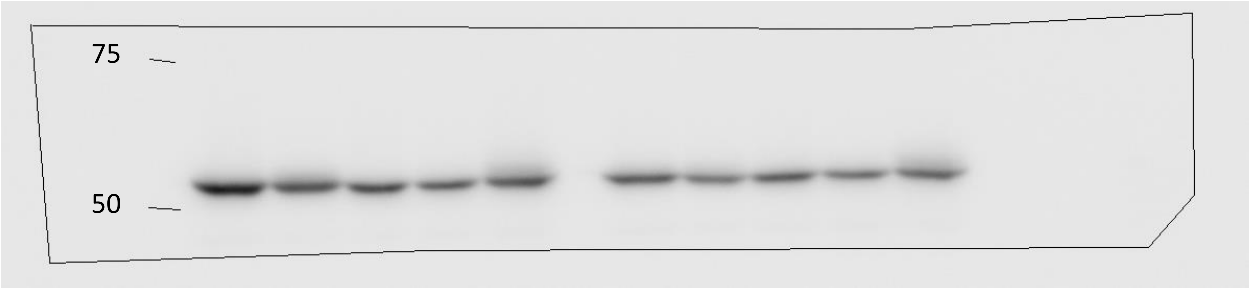

Figure 6a (continued)

GAPDH

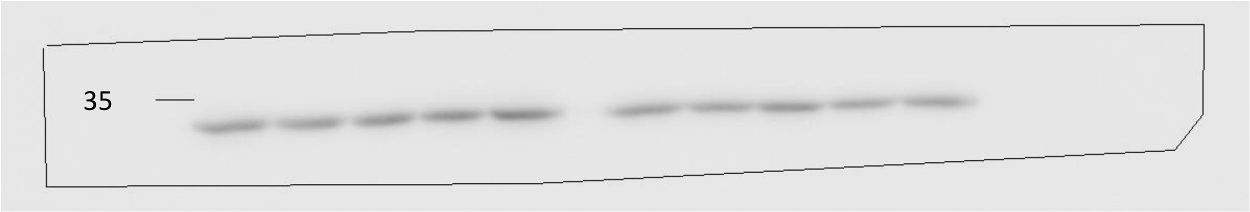

Figure 6f

POLE1

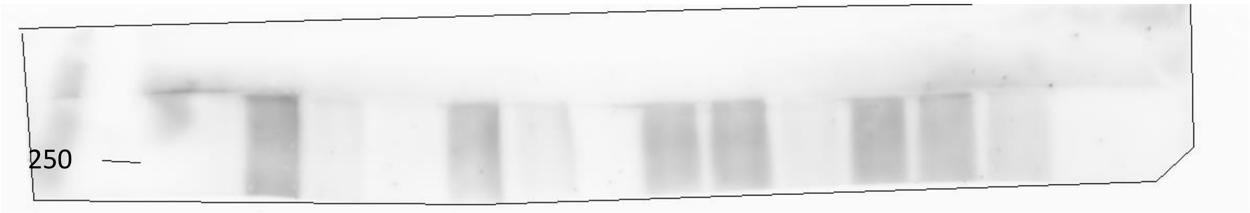

Myc

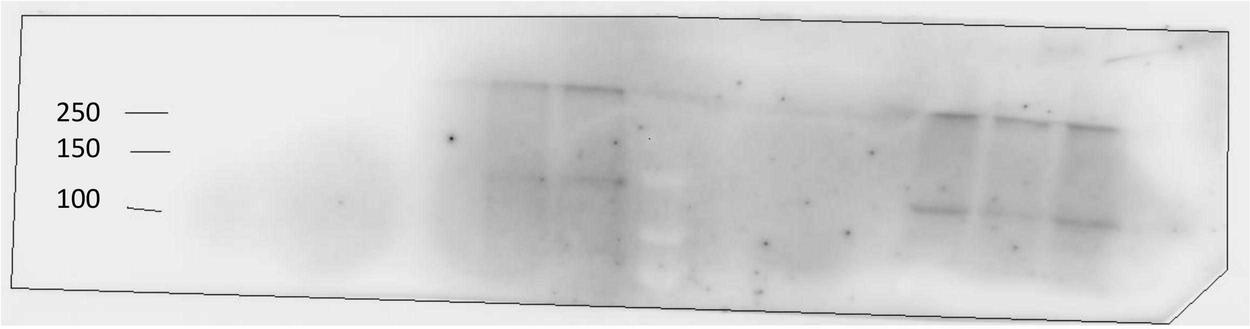

POLD1

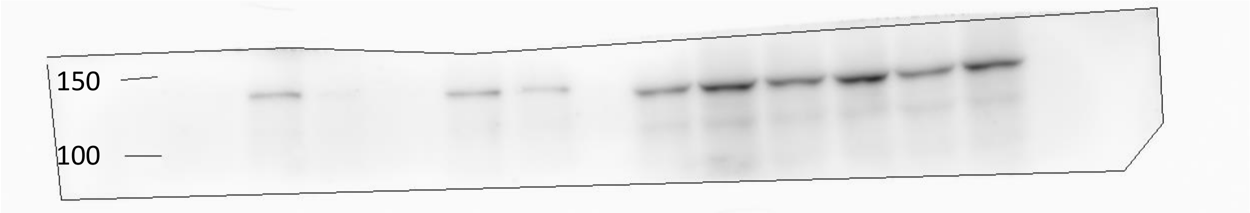

POLE2

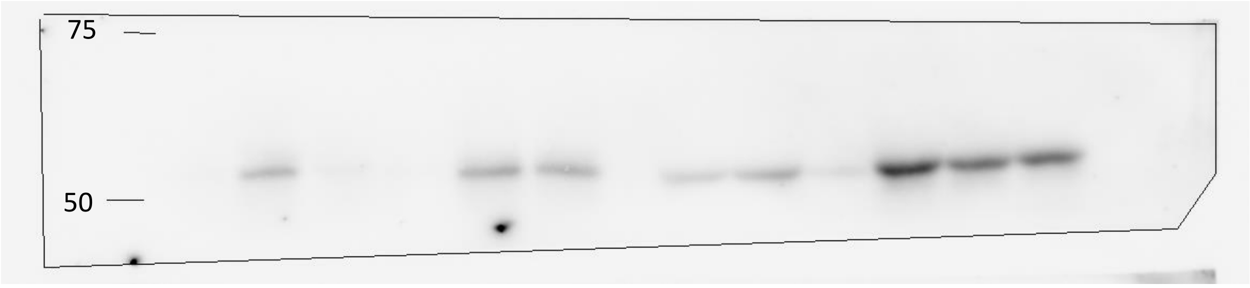

H3

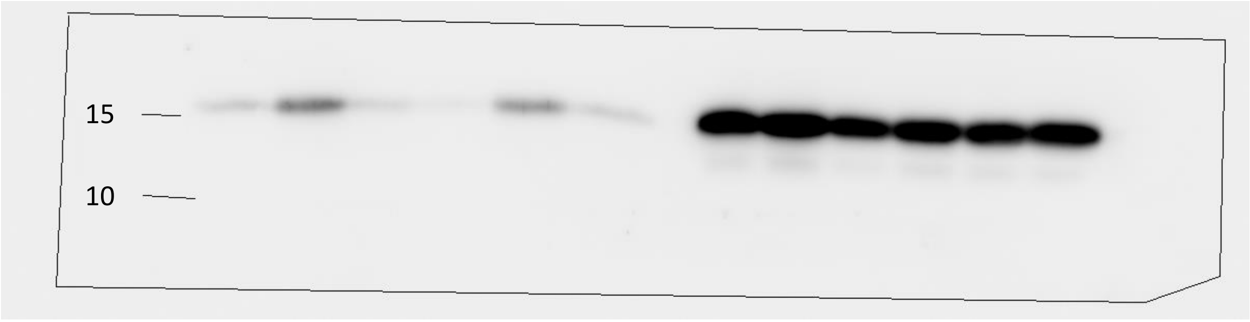

Supplementary figure 6 (only the first 5 lanes are presented on the figure)

POLE1

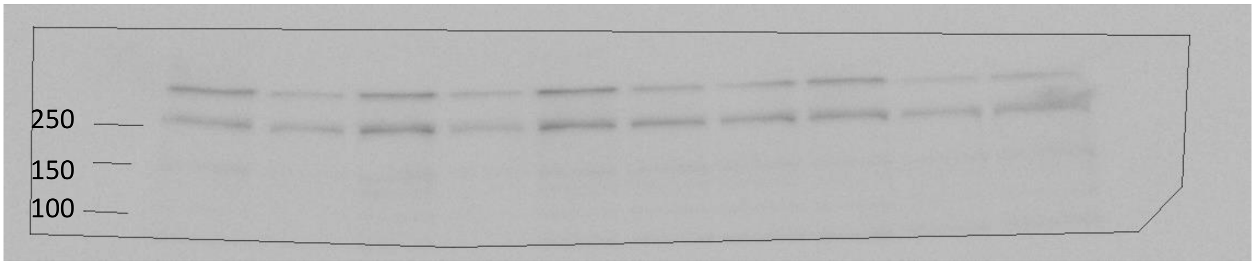

Myc

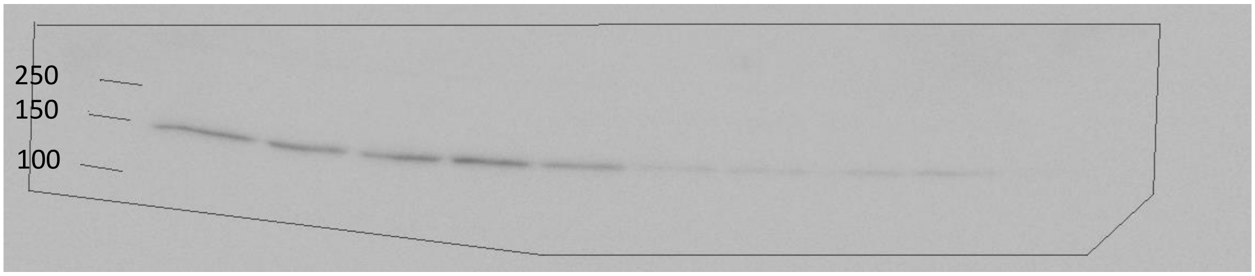

POLE2

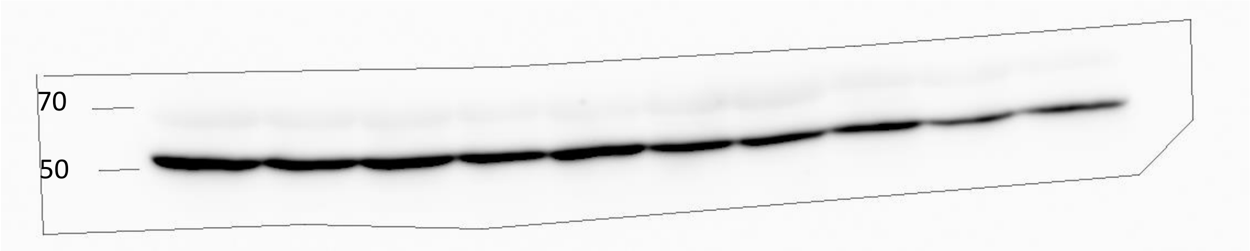

pCHK1

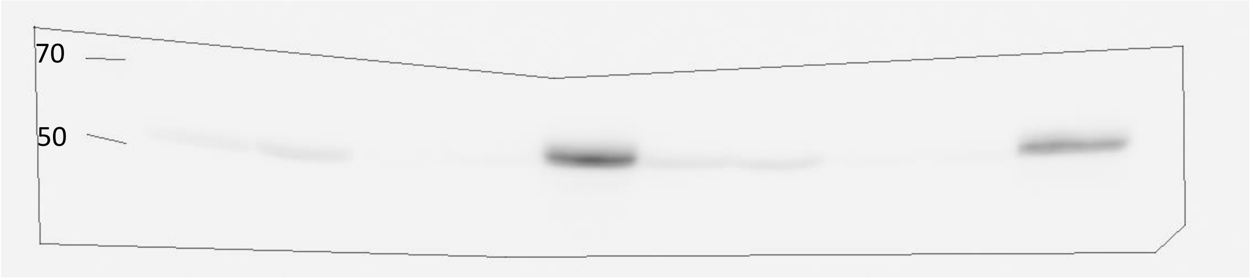

CHK1

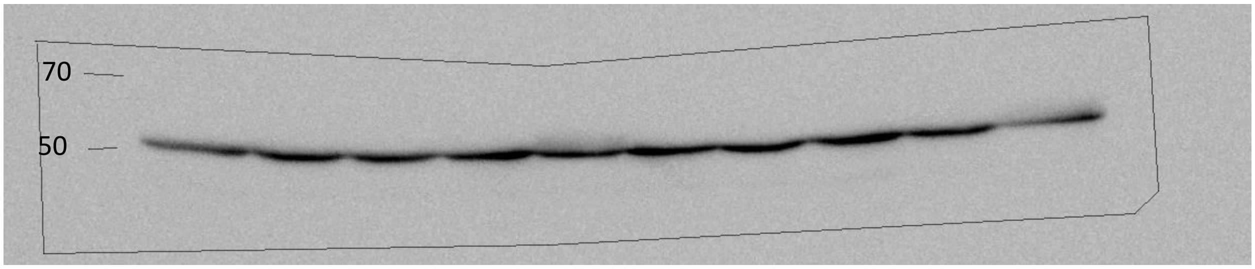

Supplementary figure 6a (continued)

GAPDH

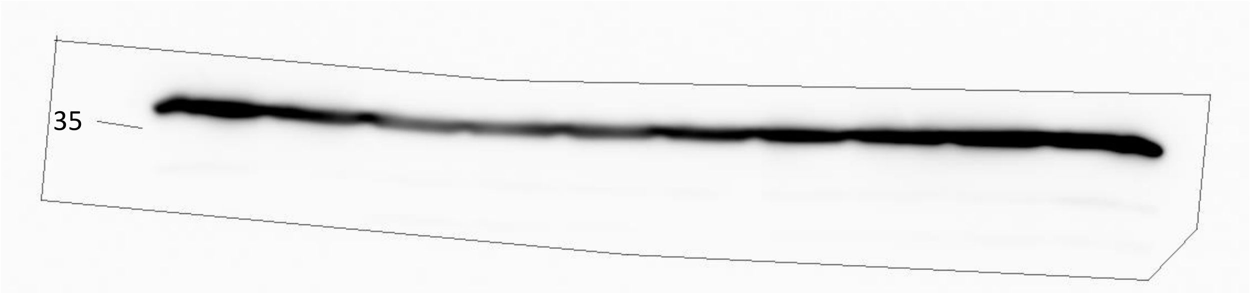

Supplement: Supplementary file 6 — Source Data [file 41467_2022_34911_MOESM6_ESM.zip › Source_data_uncropped_images.pdf]
